# A novel SadP-scFv UCHT1 lectibody activates T cells and mediates lysis of Burkitt's lymphoma cells

**DOI:** 10.1039/d5cb00027k

**Published:** 2025-11-20

**Authors:** Jana Tomisch, Jonas Gräber, Olga N. Makshakova, Pavel Salavei, Francesca Rosato, Sarah Frisancho Mariscal, Annabelle Varrot, Anne Imberty, Winfried Römer

**Affiliations:** a Faculty of Biology, University of Freiburg 79104 Freiburg Germany winfried.roemer@bioss.uni-freiburg.de; b Signalling Research Centres BIOSS and CIBSS, University of Freiburg 79104 Freiburg Germany; c Core Facility Signalling Factory & Robotics, University of Freiburg 79104 Freiburg Germany; d Université Grenoble Alpes, CNRS, CERMAV 38000 Grenoble France

## Abstract

Abnormal glycosylation has been long considered a hallmark of cancer progression. Carbohydrate-binding proteins, also known as lectins, offer a unique way to target glycosylation changes in malignant cells. The present study repurposes SadP, a monomeric lectin from *Streptococcus suis*, to target globotriaosylceramide (Gb3), a glycosphingolipid overly abundant in many cancer types including Burkitt's lymphoma. The lectibody was designed as a fusion protein by linking the SadP to the scFv UCHT1 anti-CD3 antibody resulting in a bispecific T cell engager (BiTE)-like protein referred to as lectibody. Protein expression was carried out in *Escherichia coli* and the resulting lectibody was purified using affinity and size exclusion chromatography. The lectibody was tested for its specificity in binding Gb3-positive cancer cells by flow cytometry. T-cell-mediated cytotoxicity was measured in a bioluminescence-based cytotoxicity assay, and T-cell activation was assessed by evaluating CD69 and CD71 expression on PBMCs, incubated with target cells and the lectibody. The present study demonstrates that the monomeric and monovalent SadP-scFv UCHT1 lectibody can redirect T cell cytotoxicity towards Gb3^+^ Burkitt's lymphoma cells, resulting in a dose-dependent target cell lysis up to 65% *in vitro* at a concentration of 10 nM. In the same experimental setting, negative control cells characterized by a low or absent Gb3 content remained unaffected. Lectibody-induced T cell activation resulted in a significant increase in CD69 and CD71 surface expression in PBMCs incubated with SadP-scFv UCHT1 and Gb3 positive cancer cells. This study highlights the potential of lectins in immunotherapy for the treatment and eradication of malignant cells. The SadP-based lectibody demonstrates improved efficacy and yield when compared to the previously engineered StxB-scFv UCHT1 lectibody, therefore opening the possibility for its use in an *in vivo* model.

## Abstract 2

We describe the design and characterization of a novel SadP-scFv UCHT1 lectibody, a fusion protein consisting of the lectin SadP, which specifically binds to globotriaosylceramide, and the scFv UCHT1, recognizing the CD3 receptor on T cells.

## Graphical abstract

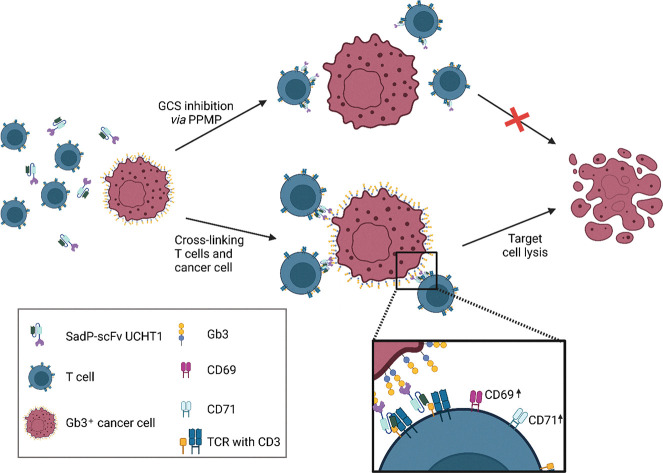

## Introduction

As one of the leading causes of death worldwide, cancer accounted for 10 million deaths in 2020 according to the WHO.^1^ It is mainly caused by genetic and epigenetic changes. Conventional treatment methods such as surgery, radio- and chemotherapy often reach their limits in clearing cancer completely while sparing healthy cells.^2–4^ The need for improved, on-target treatments led to the fast arise of immunotherapy over the last decades. Therapies harnessing the patient's immune system entail the administration of cancer vaccines or antibody treatments with clinical success in treating a variety of malignancies.^5,6^ Typically, monoclonal antibodies (mAbs), bispecific antibodies (bsAbs) or antibody–drug conjugates (ADCs) are directed against specific tumour-associated antigens (TAA).^7,8^ Among others, bispecific T cell engagers (BiTEs) represent a class of bsAbs that is of particular relevance. BiTEs are fusion proteins that usually consist of two single chain variable fragments (scFvs) fused with a linker. The first one is typically an anti-CD3 scFv that recognizes and engages one or more chains of the CD3 receptor associated with the T cell receptor (TCR). The second scFv displays specificity towards a TAA at the surface of the cancer cell.^9^ The best-known example of a clinically applied BiTE is Blinatumomab (MT103). Blinatumomab is the first FDA-approved BiTE with specificity for CD19 on the surface of B cells.^10,11^ This first-in-class BiTE is indicated for use in patients with relapsed and/or refractory (R/R) non-Hodkin lymphoma and R/R B cell acute lymphoblastic leukaemia (B precursor ALL).^10,11^ Blinatumomab showed a 3-year overall survival (OS) rate of 85% with recurrence-free survival (RFS) being 80%, with the patients under 55 years of age benefitting most.^12^ Since Blinatumomab's approval in 2014, several BiTEs have been evaluated in clinical trials and many more are in development. Some examples include (i) the AMG330 anti-CD33 BiTE to treat patients with R/R acute myeloid leukaemia (AML),^13^ (ii) the Pasotuxizumab (AMG 212) for treatment of patients with metastatic castration-resistant prostate cancer,^14^ and (iii) the Solitumomab targeting epithelial cell adhesion molecule (EpCAM) in patients with R/R advanced stage solid cancers.^15^

The therapeutic effect exerted by the BiTE is the result of a simultaneous engagement of lymphocytes and cancer cells, followed by cell-to-cell cross-linking and the formation of an immunological synapse that triggers T cells’ cytotoxic signalling. T cell activation is likely a consequence of multiple BiTE-induced clustering of CD3 complexes in a major histocompatibility complex I (MHC I) independent manner, without the necessity for co-stimulatory signals.^16^ T cell activation is a tightly controlled cascade that follows concise time and expression patterns, leading to an activation of protein kinase C and a rise of intracellular Ca^2+^.^17–20^ The signalling cascade set into motion upon activation ultimately results in the release of certain cytokines like IFN-gamma, and IL-2,^21^ the emission of granzymes and perforin and the upregulation of activation markers such as CD69, CD71, CD25 or HLA.^22–25^

Identifying a target antigen, specific to the cancer cell remains a major challenge in the development of new immunotherapies. Over the past decade, cancer research has highlighted the impact of changes in post-translational processes, alongside genetic and epigenetic alterations, on cancer occurrence. Progress in the field of glycobiology has provided evidence to link altered glycosylation patterns to malignant progression. Consequently, glycoproteins or glycans represent the highest number of FDA-approved biomarkers for cancer detection.^26,27^ Altered glycan signatures in cancer include truncated or overly branched versions of glycans, the appearance of neoantigens, overly sialylated glycans and the overall absence of certain structures. Taken together, these aberrant glycosylations are commonly referred to as, tumour-associated carbohydrate antigens (TACAs). TACAs are involved in key processes of tumour progression such as proliferation, invasion angiogenesis, the evasion of growth suppressors, the onset of metastasis and the escape from immune surveillance.^28–32^ Therefore, TACAs are well-suited targets for either diagnostic tools in cancer recognition or in immunotherapeutic approaches.^27,33^ Although TACAs represent a promising target for immunotherapy, current therapeutic approaches face difficulties in overcoming numerous challenges. For instance, carbohydrate antigens exhibit low immunogenicity, making recognition by antibodies and antibody-based treatments particularly difficult.^34^ Nonetheless, a few bsAbs made it to clinical trials. One example would be the anti-GD2 mAb called hu14.18K322A, which is currently being investigated in a phase II clinical trial for the treatment of children with newly diagnosed high-risk neuroblastoma.^35–37^ Another example is the anti-fucosyl GM1 mAb BMS-986012, which is being evaluated as a mono- or combinational therapy for R/R small cell lung cancer.^38,39^

A growing number of TACAs is represented by altered glycosphingolipids (GSL), located in the outer leaflet of the plasma membrane,^40,41^ with one of its members being globotriaosylceramide (Gb3). Gb3 is a globoside consisting of a ceramide backbone and a neutral trisaccharide head group. The ceramide is comprised of a sphingosine and a fatty acyl chain of varying length, hydroxylation and saturation.^42,43^ The trisaccharide group is composed by two galactose and one glucose moiety attached to the ceramide (Galα1–4Galβ1–4Glcβ1–Cer). Gb3 is present on germinal centre (GC) B cells where it is known as CD77 and on erythrocytes as the p^k^ blood group antigen.^28,44^ A recent study demonstrated, that Gb3 is essential in GC B cells for the production of high-affinity antibodies.^45^ Gb3 is overly abundant across many different types of cancer, such as breast-,^46,47^ colon-,^48,49^ ovarian cancer^50^ and Burkitt's lymphoma.^51,52^ Gb3 has also been widely investigated in the context of its role as receptor for pathogens that use lectins or toxins to bind to the cell surface of host cells. It is the well-known receptor for lectins from pathogens such as Shiga toxin (Stx) from *Shigella dysenteriae* and enterotoxic *Escherichia coli*, LecA from *Pseudomonas aeruginosa*, and the *Streptococcus suis* adhesion protein SadP, but also for the non-pathogenic, engineered lectin Mitsuba modelled after the *Mytilus galloprovincialis* lectin MytiLec.^53–55^ In their turn, lectins fine tune their visual affinity (referred to as avidity) through multiplying the number of binding sites, so called multivalency. The interaction in lectins with their respective glycan receptors is known to be influenced by the exposition of the carbohydrate, which is dependent on the level of saturation and chain length of the fatty acyl chain as well as the lipid environment, and their mutual orientation, as well as the amino acids of the binding sites.^56^ However, to which extent the valency is important in immunotherapeutic applications is still to be unveiled. Understanding of that will help designing new constructs with improved properties.

In recent years, therapeutic approaches have been developed that exploit the ability of lectins to bind specifically to Gb3. One approach has been to genetically engineer T cells to express a chimeric antigen receptor with lectins such as StxB, LecA or Mitsuba as target antigen recognition domains evaluating the different lectins in their suitability for a use in immunotherapeutic approaches.^57^ On the one hand, two approaches have been developed that exploit the ability of the B subunit of Shiga toxin (StxB) to bind specifically to Gb3. StxB was either genetically linked to a scFv anti-CD3^58^ or chemically bound to the scFv using non-canonical amino acids.^59^ These constructs were then called lectibodies. These studies revealed that when utilizing lectins with an increased valency, the lectibodies showed improved efficacy. However, the creation of a multimer can be difficult, therefore it could be advantageous to utilize a monomeric lectin.

SadP (Streptococcal adhesin P), also referred to as Fhb (Factor H binding protein), is a ∼200 kDa LPNTG-anchored cell wall protein that acts as a virulence factor in *S. suis* infection in piglets and more rarely in humans.^60–62^ The lectin (called SadP in this manuscript) consists of approximately 220 amino acids at the N-terminus and is made up of three α helices and 10 β-strands (β1–β10), forming a β-sandwich core domain.^60^ It contains one binding site, specific to the glycosphingolipid Gb3 with an affinity of 13 µM for the trisaccharide.^53,56,60^ It also binds to other Galα1–4βGal-containing oligosaccharides, like Gb4 or the P1 blood group antigen (GalNAcβ1–3Galα1–4Gal), although with lower affinity.^61^ Interaction occurs through hydrogen bonds with 4″-, 6″-, 2′- or 3′-hydroxyls of Galα1–4Gal moiety, while the glucose moiety is not critical for binding.^63^ However, binding of SadP to its disaccharide receptor is stronger if the glucose is present.^64^ SadP binding to Gb3 is necessary for *S. suis* to cross the blood–brain barrier (BBB) and access the central nervous system of the host.^60,61^ The attachment of *S. suis* to the host cells through the binding of SadP to its disaccharide receptor prevents the bacterium from being flushed away with the bloodstream.^65^

The present study proposes the use of the monomeric, Gb3-binding lectin domain SadP in an immunotherapy approach.^66^ As previous attempts in creating a purely monomeric lectibody were unsuccessful, a strategy based on a naturally monomeric lectin could be the solution. The authors successfully developed a SadP-based lectibody as a follow-up study to the StxB-based lectibodies previously published^58,59^ exhibiting improved efficacy, despite reduced valency. The SadP sequence was genetically linked to the UCHT1 anti-CD3 scFv. The resulting fusion protein was able to cross-link T cells with Gb3^+^ cancer cells. This specific cross-linking with a high affinity anti-CD3 promoted an activation state of the T cells. Activated T cells induced subsequent targeted lysis of Burkitt's lymphoma cells at low nanomolar concentrations.

## Materials and methods

### Antibodies and chemicals

The following antibodies were acquired from BioLegend (San Diego, CA, USA): AlexaFluor™647 anti-His-Tag (Cat. No.: 652513), FITC anti-human CD3 (Cat. No.: 317305), FITC anti-human CD71 (Cat. No.: 334103), AlexaFluor™647 anti-human CD3 (Cat. No.: 300322). The horse-anti-mouse HRP antibody (Cat. No.: 7076) was purchased from Cell Signaling Technology (Danvers, MA, USA) and the APC anti-human CD69 (Cat. No.: MHCD6905) was bought from Thermo Fisher Scientific (Waltham, MA, USA). The restriction enzyme BsaI-HF v2 was purchased from New England Biolabs (Ipswich, MA, USA). The SadP lectin was produced in-house as described in the purification section below.

Fetal bovine serum (FBS), Roswell Park Memorial Institute 1640 medium (RPMI 1640), HEPES and l-glutamine were commercially purchased from Thermo Fisher Scientific (Waltham, MA, USA). DMSO, Isopropyl-β-d-thiogalactopyranoside (IPTG), penicillin/streptomycin (P/S), kanamycin, lysogeny broth (LB), β-mercaptoethanol, l-Glutamine were purchased from Carl Roth GmbH (Karlsruhe, Germany). Pancoll was obtained from PAN Biotech (Aidenbach, Germany). Trypsin and PBS were acquired from anprotec (Bruckberg, Germany). d-Luciferin Firefly was obtained from Biosynth (Staad, Switzerland).

### Cell lines

In this study, Burkitt's lymphoma-derived cell lines Ramos and Namalwa were used (Ramos cells, ACC 603, and Namalwa: CSN/70, ACC 70, DSMZ-German Collection of Microorganisms and Cell Cultures GmbH, Germany). Ramos and Namalwa cells were maintained at 37 °C and 5% CO_2_ in RPMI 1640 containing 10% heat-inactivated FBS, 5 µg mL^−1^ P/S, and 2 mM l-glutamine.

### Isolation of PBMCs

PBMCs were isolated from healthy donors by means of, a density gradient centrifugation as described in previously published work.^58^ After centrifugation, the layer containing the PBMCs was collected from the tubes and washed with PBS. They were spun down at 398 × *g* for 10 minutes. This washing step was repeated once. If the pellet had many red blood cells left, the pellet was treated with 1 mL ammonium–chloride–potassium (ACK) lysis buffer, for 4 minutes. After that the cells were pelleted by another centrifugation step for 5 minutes at 398 × *g*. The cells were counted and then frozen at a density of 2 × 10^7^ cells per mL in FBS containing 10% DMSO and placed in liquid nitrogen for long-time storage. After thawing, PBMCs were cultivated in RPMI 1640 containing 10% FBS, 2.5 µg mL^−1^ P/S, 1% HEPES and 0.0035% β-mercaptoethanol at 37 °C and 5% CO_2_.

### Homology modelling of the lectibody structure

The structure of the SadP-scFv UCHT1 fusion protein was built up using Modeller9.15 (San Francisco, CA, USA).^67^ Diabody 31 (PDB code: 6KR0) was used as a template for the anti-CD3 scFv (as described in ref. 58). The model of SadP was obtained from the X-ray structure of SadP (PDB code: 5BOA).

### Protein–protein docking for monomeric lectibody design

The structure of SadP and the homology-based structure of scFv were used for protein–protein docking. The docking was performed using the ClusPro web server.^68,69^ The top ten docking poses were selected for the analysis.

### Structure optimization

The spatial structure of the whole fusion protein was built up retaining the SadP and scFv mutual orientation selected from docking results. The linker was added using Modeller9.15 (San Francisco, CA, USA). The structure was equilibrated using molecular dynamics (MD). MD simulations were carried out in the Amber22^70^ using the Amber14SB force-field parameters. The fusion protein was immersed into a water box with periodic boundary conditions. The TIP3P model was used for water molecules. Three chloride ions were added to neutralize the charge of the protein. An integration step of 2 fs was used together with the SHAKE algorithm constraining the bonds involving hydrogen atoms.^71^ The Particle Mesh Ewald (PME) method was used for long-ranged electrostatic interactions.^72^

The simulations were carried out in the isothermal isobar thermodynamic ensemble at 300 K. The temperature and the pressure were kept constant using a Langevin thermostat with a collision frequency of 2 ps^−1^ and a weak coupling algorithm with a relaxation time of 2 ps, respectively. First, the system was minimized for 5000 steps and then equilibrated. In the production run, 1000 ns of the trajectory were accumulated. The molecular mechanics Poisson–Boltzmann surface area (MM–PBSA) analysis was performed on the last 200 ns.

### Cloning and transformation of the SadP-scFv UCHT1 lectibody

The plasmids used to express the SadP-scFv UCHT1 lectibody was based on the pET30a_Cer (kindly provided from enGenes Biotech GmbH, Vienna, Austria).^73^ For the cloning of the SadP-scFv UCHT1 lectibody, the DNA insert was custom-ordered from Integrated DNA Technologies (IDT; Coralville, IA, USA; Fig. S1a). The BsaI cutting sites to the insert were added *via* PCR using the primers 5′-AGG̲T̲C̲T̲C̲TTACACATATGAAACAGCAATCACC-3′ and 5′-AG̲T̲C̲T̲C̲TGTGCGTC-TTTTCTTTCTCAAG-3′. The BsaI sites were also added to the vector (5′-AG̲G̲T̲C̲T̲C̲TTGTAGCACTAGCAGAGAAGGC-3′ and 5′-G̲G̲T̲C̲T̲C̲TTCACTAAATTCGAACGCCAGCAC-3′). Following amplification, insert and backbone were digested using BsaI-HF v2 and ligated *via* T4 ligase according to the manufacturer's protocol. Successfully ligated plasmids were then transformed into NEB5-alpha *E. coli* (New England Biolabs, Frankfurt a.M., Germany), regenerated and plated on LB agar plates. Plasmids from kanamycin-resistant clones were purified using the Monarch MiniPrep Kit from NEB and then sequenced (Eurofins, Ebersberg, Germany) using the standard T7 and T7 term primers. The pET30a (SadP-scFv UCHT1)_Cer was consecutively transformed into BL21(DE3) *E. coli* (New England Biolabs, Frankfurt a.M., Germany) to enable protein production (plasmidmap can be found in Fig. S1b).

### Protein expression optimization

To attain the optimal protein yield, the ideal combination of expression temperature and amount of IPTG to induce protein expression was determined. To that end, 30 mL LB-medium containing kanamycin (50 µg mL^−1^) were inoculated with BL21(DE3) cells containing the pET30a (SadP-scFv UCHT1) Cer. The bacteria were cultured at 37 °C while continuously shaking (200 rpm), and once the culture reached an optical density (OD) of 0.8, protein expression was induced with either 0.1, 0.5 or 1 mM of IPTG. The samples with different IPTG contents were then moved to different incubators so that each IPTG concentration was used in combination with one of the expression temperatures (20 °C, 25 °C or 30 °C). When the expression time was over (3 hours for 30 °C, 5 hours for 25 °C and 18 hours for 20 °C) the samples were spun down at 4790 × *g* for 15 minutes at 4 °C and resuspended in 5 mL lysis buffer per gram pellet (PBS, 10 mM Imidazole, 20 µg mL^−1^ DNAse, 1 mM MgCl_2_). The mixture was incubated at 4 °C for 30 minutes while continuously rotating. Following that, the solution was sonicated on ice for three cycles (60% power, 50% cycle, 30 seconds on, 1 minute off). The lysate was then spun down at 20 000 × *g* for 30 minutes at 4 °C and the supernatant was filtered with a 0.45 µm filter syringe. For SDS-PAGE and immunoblot analysis the lysates were then frozen.

### Big batch protein expression and purification

One day prior to protein expression, a 30 mL pre-culture of LB-Medium containing kanamycin was started with transformed BL21(DE3) *E. coli*. The pre-culture was incubated at 220 rpm, overnight (ON; 18 hours) at 37 °C. Then three litres of LB-medium containing kanamycin were inoculated with 10 mL pre-culture and incubated at 37 °C at 180 rpm until the OD reached 0.8. Subsequently, protein expression was induced with 1 mM IPTG, and the temperature was lowered to 20 °C. Protein expression was performed for 18 hours, while continuously shaking at 140 rpm. Afterwards, the cultures were centrifuged at 4790 × *g* for 15 minutes at 4 °C. The pellet was lysed and processed as described above. The supernatant was recovered and purified by immobilized metal affinity chromatography (IMAC; binding buffer: PBS, 10 mM Imidazole, 150 mM NaCl; elution buffer: PBS, 500 mM Imidazole) on a 5 mL Histrap column using an ÄKTA avant™ chromatography system (Cytiva, Marlborough, MA, USA). Immediately following the affinity purification, a size exclusion chromatography (SEC) step was added using a HiLoadTM 26/600 SuperdexTM 200 column (Cytiva, Marlborough, MA, USA). Buffer exchange to the protein storage buffer (HBS buffer, 20 mM HEPES pH 7.4, 150 mM NaCl) also occurred in this step. The fractions containing the protein were pooled and concentrated using the Pierce™ concentrator columns (Thermo Fisher Scientific, Waltham, MA, USA). As the protein showed degradation when stored at 4 °C, 10% sucrose was added prior to snap freezing and storage at −80 °C.

### SDS–PAGE and immunoblotting

To validate the calculated protein size, an SDS–PAGE gel electrophoresis with subsequent immunoblotting was prepared. The proteins (3 µg) were mixed with reducing sample buffer (4×) (2 mL β-mercaptoethanol, 0.8 g SDS, 2 mL 0.5 M trizma base, 0.4% bromophenol, 4 mL glycerol, 2 mL ddH_2_O) and boiled for 5 minutes at 95 °C. The samples were loaded onto a 12% acrylamide gel and run in a PowerPac™Basic Power Supply (Bio-Rad Laboratories, Hercules, CA, USA) for 10 minutes at 100 V. The voltage was then increased to 130 V for 45 minutes. Afterwards, the proteins were transferred to a nitrocellulose membrane with a current of 0.18 mA per gel, for 30 minutes (peqPOWER 250 Volt Power Supply, VWR International, Radnor, PA, USA). The membrane was blocked with 3% BSA in TBS-T for 1 hour. The proteins were then labelled overnight at 4 °C, using an anti-6× His-Tag antibody (1:1000 in blocking buffer) while continuously shaking. After incubation with the primary antibody the membrane was washed three times with TBS-T for 5 minutes. The secondary antibody (horse-anti-mouse HRP) was diluted 1:2000 in blocking buffer and incubated for 60 minutes at RT while continuously shaking. The membrane was subsequently washed three times with TBS-T for 5 minutes each and luminescence was detected using a Novex® Electrochemiluminescence kit (Invitrogen AG, Carlsbad, CA, USA). Images were acquired using a FUSION FX imager and the FusionCapt Advance Solo software v.17.04a (Vilber Lourmat Deutschland GmbH, Eberhardzell, Germany).

### Flow cytometry

The binding of the SadP-scFv UCHT1 lectibody to target and effector cells was assessed *via* flow cytometry. To this end, 2 × 10^5^ cells per well were plated in a 96-well U-bottom plate. The cells were spun down for 3 minutes at 1600 × *g* on 4 °C (these settings were kept constant for all following centrifugation steps). The cells were then washed once with FACS buffer (PBS supplemented with 3% FBS) and spun down. The cells were then resuspended in FACS buffer containing the SadP-scFv UCHT1 lectibodies and incubated on ice for 30 minutes. Afterwards the cells were washed once, and the protein was detected using an AlexaFluor™647 (AF647) anti-6× His-Tag antibody (1:1000) and incubated for 20 minutes at 4 °C, in the absence of light. Subsequently, the cells were washed once more and resuspended in FACS buffer. Finally, samples were then analysed with the Attune NxT flow cytometer (Thermo Fisher Scientific, Waltham, MA, USA) and the data was analysed with the FlowJo software (V.10.05.3; Becton Dickinson, Franklin Lakes, NJ, USA).

### Depletion of glucosylceramide-based glycosphingolipids by PPMP treatment

To deplete Ramos cells of globotriaosylceramide, 2 × 10^5^ cells per well were seeded in a 6-well plate and cultured for 72 hours in the presence of 2 µM of the glucosylceramide synthase (GCS) inhibitor d-l-*threo*-1-phenyl-2-palmitoylamino-3-morpholino-1-propanol (PPMP). PPMP is an inhibitor for the synthesis of glucosylceramide-based GSLs.^74^ The absence of Gb3 on the surface of Ramos cells was measured by flow cytometry by incubating them with 2.6 nM StxB-AF647 and 20 nM SadP, subsequently detected using an anti-His-Tag-AF647 antibody.

### Cytotoxicity assay

A bioluminescence-based cytotoxicity assay was employed to determine the ability of the SadP-scFv UCHT1 lectibody to induce T cell mediated target cell lysis in Burkitt's lymphoma cells. To this end, 1 × 10^4^ luciferase expressing tumour cells (Ramos, Namalwa) per well were plated in triplicates in a white polystyrene, flat-bottom 96-well plate in medium containing 75 µg mL^−1^ luciferin. PBMCs were added to target cells in an effector-to-target ratio 5 : 1 (E : T). The SadP-scFv UCHT1 lectibody was added to the corresponding wells in concentrations ranging from 0.2 nM to 10 nM. To normalize for spontaneous cell death, well containing (1) only target cells, or (2) target and effector cells in absence of lectibody were added as controls. Maximum cell death was recorded by adding 2% Triton X-100 to target cells. All samples were incubated for a total of 48 hours and luminescence was measured at 7 hours, 24 hours and 48 hours. The percentage of specific killing was calculated by using the following formula:
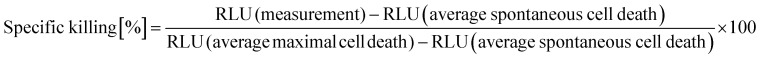


In addition, the cytotoxicity of the SadP-scFv UCHT1 lectibody against Ramos cells was compared with that of a Shiga toxin B-subunit (StxB)-scFv UCHT1 lectibody. For this purpose, the cells were treated as described above and lectibody concentrations in the range of 1 nM to 100 nM were used.

The measured data was analysed using GraphPad Prism (V.8.4.3; GraphPad Software Inc., San Diego, CA, USA).

### Activation of T cells

To assess T cell activation in the presence of target cells and the SadP-scFv UCHT1 lectibody, effector cells (PBMCs) and target cells were co-cultured in a 5 : 1 (E : T) ratio in presence (Sample) or absence (Ctrl (E + T)) of SadP-scFv UCHT1. Ramos or Namalwa cells (6 × 10^4^) were incubated with 3 × 10^5^ PBMCs at 37 °C under 5% CO_2_ for 18 hours, 24 hours or 48 hours. Cells were treated with 5 or 10 nM SadP-scFv UCHT1 or left untreated in PBS-containing medium. Untreated PBMCs were used as an antibody control (UTD Ab Ctrl) to ensure the fold increase was calculated without antibody background.

The plate was then spun down at 1600 × *g* for 3 minutes at 4 °C, washed once with FACS buffer and stained with an antibody mix (either anti-CD3/anti-CD69 or anti-CD3/anti-CD71) for 20 minutes at 4 °C. After incubation, the cells were centrifuged, washed and resuspended in FACS buffer. The fluorescence intensity of treated cells was measured using the Attune NxT flow cytometer (Thermo Fisher Scientific, Waltham, MA, USA) and the data was analysed with the FlowJo software (V.10.05.3; Becton Dickinson, Franklin Lakes, NJ, USA). The mean fluorescence intensity (MFI) of each sample was determined. The fold increase was calculated as follows:
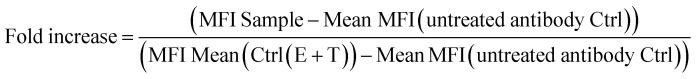


### Cell viability (MTT) assay

To exclude T cell-independent cytotoxicity of SadP-scFv UCHT1, Ramos and Namalwa cells were treated with increasing concentrations of the lectibody for 24 hours. Cells were seeded in a density of 3 × 10^4^ cells per well in a flat-bottom, standard 96-well plate and treated with a wide range of concentrations of the SadP-scFv UCHT1 (0.1–50 nM) in a final volume 100 µL. At 24 hours post-incubation at 37 °C under 5% CO_2_, 10 µL of MTT labelling solution (MTT Cell Proliferation Kit, Roche Holding, Basel, Switzerland) were added to each well and the plate was incubated for 4 hours at 37 °C. Then, 100 µL of the solubilisation reagent was added to each well and the plate was incubated at 37 °C overnight. Cells which are metabolically active are able to take up the yellow tetrazolium salt (MTT) and reduce it to the purple formazan, which is then exocytosed. The amount of formazan in the medium can be measured after solubilisation by its absorbance. The absorbance of the samples was measured at 550 nm using a BioTek microplate reader (BioTek Instruments Inc., Winooski, VT, USA). The data were analysed further using GraphPad Prism software (V.8.4.3; GraphPad Software Inc., San Diego, CA, USA).

### Statistical analysis

To assess statistical relevance, an ordinary one-way ANOVA was performed using multiple comparison to compare the mean of each sample to that of the control. The data was corrected for multiple comparison using the Holm–Sidak test. Data was considered significant if *p* < 0.05 and the confidence level was set to 95% confidence interval. The statistical analysis was performed using GraphPad Prism software (V.8.4.3; GraphPad Software Inc., San Diego, CA, USA).

### Quantification of endotoxins

The Pierce™ Chromogenic Endotoxin Quant Kit (Cat. No.: A39552, Thermo Fisher Scientific, Waltham, MA, USA) was used to determine the endotoxin concentration of the protein solutions used. The pH value of all samples was adjusted to between 6 and 8. The lipopolysaccharide (LPS) concentration was detected in the samples by measuring the proteolytic activity of the proenzyme factor C from amoebocytes. This proenzyme is activated in the presence of LPS. The proteolysis of a synthetic substrate then leads to the formation of a yellow colour, which can be measured with a plate reader at an absorption of 405 nm.

In addition, endotoxin standards were measured in defined concentrations between 0.1 nM and 1 nM and used to create a standard curve. By fitting the values of the protein samples to the standard curve, the LPS concentrations were calculated.

## Results

### Design and modelling of the SadP-scFv UCHT1 lectibody

The design of our lectibody was inspired by the BiTE structure.^75–77^ BiTEs bind the CD3 receptor on T cells in a monovalent fashion and with rather low affinity.^77^ T cell activation is the result of the binding of several BiTEs to CD3 receptor complexes on the same T cell leading to CD3 receptor clustering, which in turn facilitates T cell activation.^16,78^ Only excessively abundant target antigens are therefore able to provide sufficient signals for this clustering to mediate activation.

**Fig. 1:**
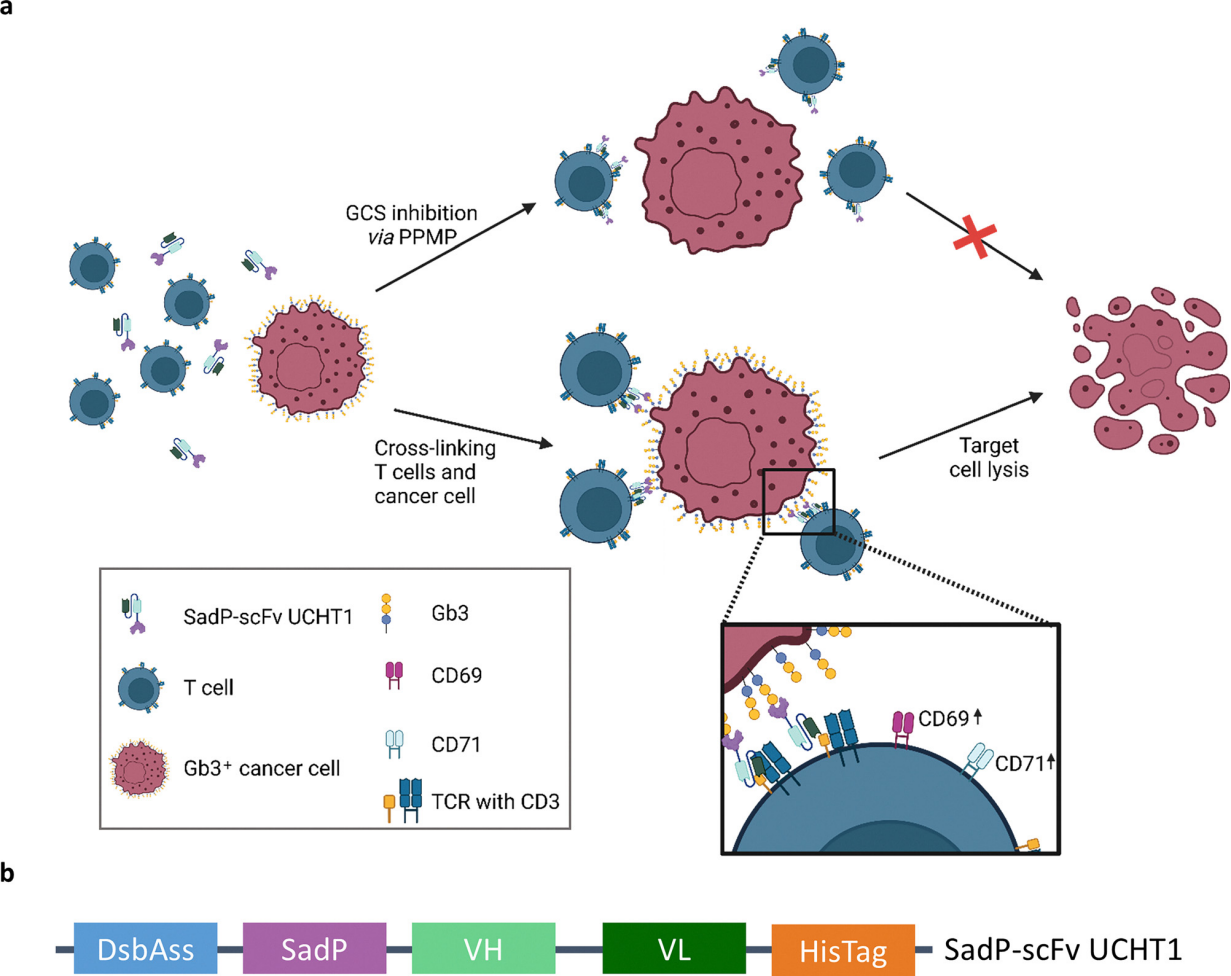
Schematic representation of the mechanism of action of SadP-scFv UCHT1, and lectibody sequence. (a) The lectibody cross-links the T cell (blue) with a cancer cell (red) *via* the overly abundant Gb3 on the surface of the latter. This cross-linking leads to T cell activation measured by an increase of CD69 and CD71 on the surface of the T cell. If the glucosylceramide synthase (GCS) is inhibited and the cell therefore depleted of GSLs, cross-linking is not possible and target cell killing is abolished. This figure was created in BioRender (https://BioRender.com/n21n476). (b) SadP is connected to the heavy chain (VH) of the scFV UCHT1 *via* a short (1× GGGS) linker which in turn was connected to the light chain (VL) *via* a long (4× GGGS) linker. The SadP is located at the N-terminal end of the protein. The DsbA signalling sequence (DsbAss) ensures the periplasmic export of the lectibody.

**Fig. 2:**
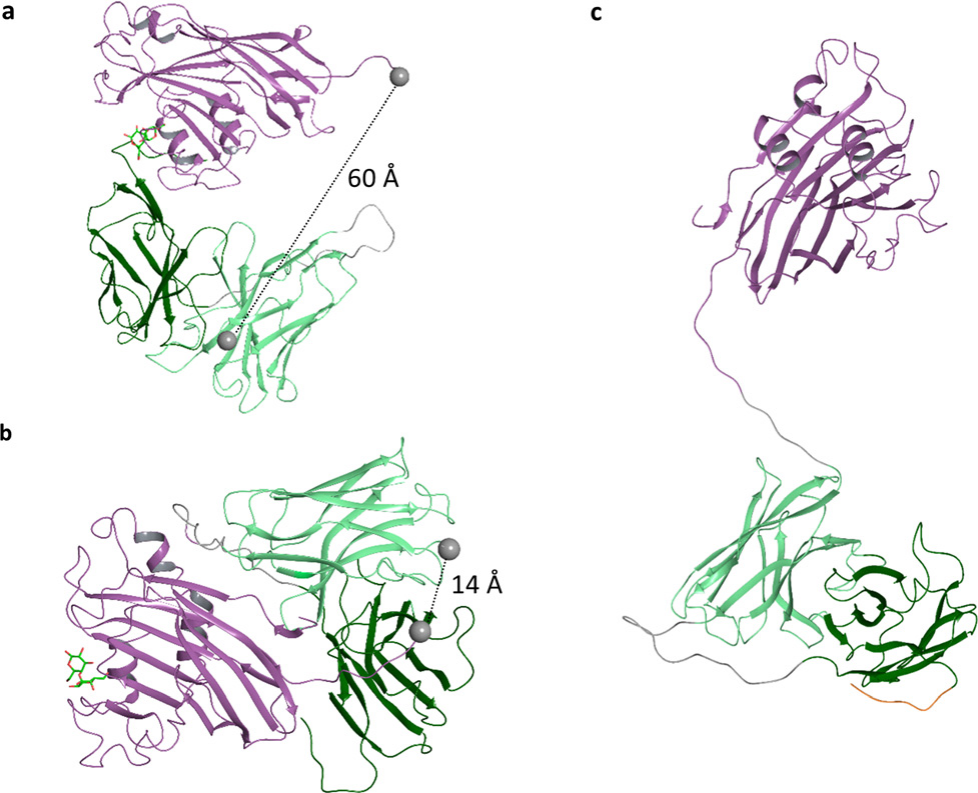
Protein–protein docking and homology-based model of SadP-scFv UCHT1. (a) Two docking poses between SadP and scFv UCHT1 proteins where the carbohydrate binding site of SadP is interfered by scFv UCHT1 and (b) free from protein–protein interactions. The d-galactose is copied from pdb: 5BOA to indicate the location of the sugar binding site coloured as green for C atoms and red for O atoms. The C alpha atoms of the C-terminus of SadP and the N-terminus of scFv UCHT1 are depicted as grey balls. (c) A homology-based model of the SadP-scFv UCHT1 lectibody. Colour coding: SadP – violet, heavy chain variable fragment – light green, light chain variable fragment – dark green, linkers – grey.

Following this approach, the lectibody was designed by exchanging the target antigen antibody with a lectin that recognizes aberrant glycosylation on the surface of cancer cells. Like BiTEs, the lectibody is designed to connect a cancer cell (Fig. 1(a), left red-coloured cell) to a T cell (Fig. 1(a), blue-coloured cell). CD3 receptor clustering would then lead to an activation of recruited T cells. The resulting activation can be measured by upregulation of CD69 and CD71 markers on the surface of the T cell (Fig. 1(a), bottom panel), followed by a lysis of the cancer cell (Fig. 1(a), right). In absence of Gb3, T cells engaged by the lectibody are not activated and target cell lysis does not occur. The design of this lectibody is similar to our previously published one, namely StxB-scFv UCHT1.^58^ In the SadP-scFv UCHT1 construct, SadP is linked by a short linker (1× GGGS) to the heavy chain (VH) of the scFv. The VH is in turn connected to the light chain (VL) of the scFv by a long linker (4× GGGS; Fig. 1(b)). The DsbA signalling sequence (DsbAss) for periplasm export, located on the N-terminal end of the fusion protein and a 6× His-Tag for protein purification is located at the C-terminus (Fig. 1(b)). As the scFv UCHT1 relies on an oxidizing environment for correct folding, the translocation into the periplasm *via* the DsbAss sequence was necessary.^79–81^ Before designing the SadP-scFv UCHT1 fusion construct we ensured that the two protein parts would have no tendency to block their respective binding sites by protein–protein interactions. Additionally, the effect of the linker length was investigated. Protein–protein docking revealed one binding pose where scFv UCHT1 is in close proximity to the carbohydrate binding site of SadP and might potentially interfere with the sugar binding. In this complex, the distance between the C-terminus of SadP and N-terminus of scFv UCHT1 was about 60 Å (Fig. 2(a)). However, regarding the flexibility of the C-terminus of SadP, this distance could be shortened down to 34 Å. Therefore, we concluded that the length of the linker should be shorter than 34 Å. The distances between the C-terminus of SadP and the N-terminus of scFv UCHT1 in other docking poses were analysed under this premise. The docking poses meeting this condition showed a distance that amounted to 14 Å (Fig. 2(b)). Consistent with this finding, the GGGS linker, also previously used for the StxB-based lectibody,^58^ was selected to connect SadP and scFv UCHT1.

As the next step of modelling, the full structure of the fusion protein, meaning SadP and scFv UCHT1 connected by a short (1×) GGGS linker (Fig. 2(c)), was equilibrated using MD. One of the initial structures was constructed by retaining the mutual protein orientation as shown by the docking results (Fig. 2(b)). To enhance conformational sampling, three additional structures were created, where scFv UCHT1 was systematically positioned in respect to SadP (Fig. S2). In these initial structures, the scFv UCHT1 was put at a distance from SadP that allowed the linker to freely explore the conformational space without being trapped by protein–protein interactions. In the course of MD trajectories, the two parts of the lectibody revealed a dynamic character of contacts showing association and dissociation events. Furthermore, the contact area of SadP in the lectibody sampled by MD was large (Fig. S3). The binding energies estimated for a number of binding modes revealed only slight differences (Table S1). The findings that SadP has rather no preferred mode of interaction when fused as the scFv UCHT1 are consistent with the hydrophilic nature of SadP. From a structural point of view, the results of this modelling suggest that the SadP-scFv UCHT1 lectibody connected *via* a short (1×) GGGS linker can bind both to Gb3 and to the CD3 receptor without any hindrance (Fig. S4).

### SadP-scFv UCHT1 binds specifically to Gb3 and is able to redirect T cells and mediate target cell lysis

**Fig. 3:**
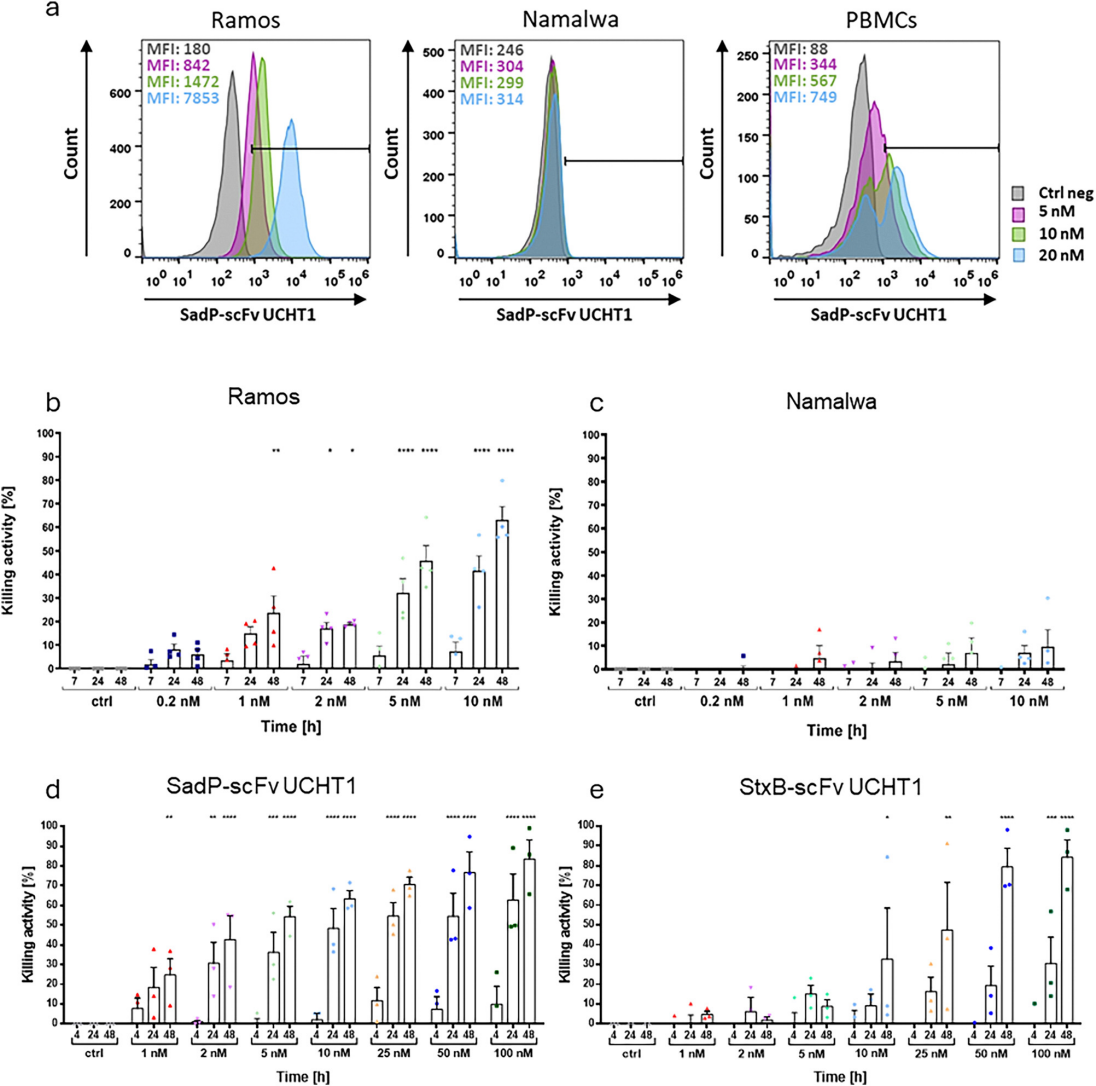
Binding and T cell-mediated target cell lysis induced by the SadP-scFv UCHT1 lectibody. (a) Representative flow cytometry histograms for target (Ramos, Namalwa) and effector (PBMCs) cells. The cells were incubated with increasing concentrations of SadP-scFv UCHT1 and stained with an anti-His-Tag-AF647 antibody to detect SadP-scFv UCHT1 at the cell surface. The SadP-scFv UCHT1 exhibited robust, dose-dependent binding to Gb3^+^ Ramos cells and PBMCs, while binding to Gb3^−^ Namalwa cells was abolished. (b) Ramos and (c) Namalwa cells were incubated together with PBMCs in a ratio of 5 : 1 (E : T) and SadP-scFv UCHT1 (0.2–10 nM) for 7 hours up to 48 hours. (b) At 24 hours post-incubation 15% of target cell lysis was recorded in presence of 1 nM of lectibody, reaching up to 42% for 10 nM. After 48 hours, the specific target cell lysis increased to 42% with 5 nM and 65% killing with 10 nM SadP-scFv UCHT1. The data are shown as the mean ± SEM (*N* = 3) of four separate experiments. *n* = 4. (c) When the Gb3^−^ Namalwa cells were incubated with PBMCs in presence of the SadP-scFv UCHT1 no significant T cell-mediated target cell lysis was registered. (d) and (e) Comparison of the killing rates of Ramos cells triggered by the lectibody SadP-scFv UCHT1 (d) and StxB-scFv UCHT1 (e) in similar concentrations. The SadP lectibody achieved higher killing rates at lower concentrations and comparable cytotoxicity values to the StxB lectibody at higher concentrations. The data are shown as the mean ± SEM (*N* = 3) of three separate experiments. *n* = 3. The experiments were performed with PBMCs derived from 3 different donors (*N* = 3). Each dot represents the averaged data from each individual donor. Statistical significance was evaluated using a one-way ANOVA. *p*-values < 0.5 were considered significant. *p ≤ 0.05; **p ≤ 0.01; ***p ≤ 0.001; ****p ≤ 0.0001.

SadP-scFv UCHT1 was cloned into the pET30a_Cer vector and expression was performed in *E. coli.* To determine the optimal conditions for achieving the highest yield, different combinations of expression temperature and IPTG concentration were tested (Fig. S5). A temperature of 20 °C and an overnight induction of protein expression with 1 mM IPTG proved to be optimal. The lectibody was purified by IMAC utilizing the 6× His-Tag, and subsequent SEC with simultaneous buffer exchange (Fig. S6a–c). Afterwards, the proteins were concentrated. Following purification, the lectibody was analysed *via* SDS-PAGE and subsequent immunoblotting where the calculated size of the SadP-scFv UCHT1 of ∼52 kDa could be confirmed and the lectibody proved to be pure (Fig. S6d). A major challenge in developing novel immune agents is represented by ensuring specificity towards the target antigen to forgo unwanted side effects. Given the approval of conventional BiTE therapies for relapsed or refractory haematological cancers, Burkitt's lymphoma-derived cell lines were employed as the model in this research. To ensure specificity of the SadP-scFv UCHT1 for the target antigen Gb3 the fusion protein was incubated with cells showing a high Gb3 abundance (Ramos, termed Gb3^+^) at the surface, and cells characterized by a very low abundance of Gb3 (Namalwa, termed Gb3^−^) representing a negative control. The SadP-scFv UCHT1 was incubated with target (Ramos, Namalwa) cells and PBMCs at the indicated concentrations for 30 minutes on ice. The lectibody was then detected with an anti-His-Tag-AF647 antibody, and the samples were imaged using flow cytometry (Fig. 3(a)). While the SadP-scFv UCHT1 bound to Gb3^+^ Ramos cells and CD3^+^ PBMCs (53–100% and 18–53%, respectively), it only exhibited minimal binding to Gb3^−^ Namalwa cells (1%, Fig. 3(a)). The SadP lectin alone was found to bind to Gb3^+^ Ramos cells at concentrations between 20 nM and 208 nM, while it was not detected at the surface of Gb3^−^ Namalwa cells and PBMCs (Fig. S7).

To assess the ability of the SadP-scFv UCHT1 to induce T cell-mediated target cell lysis, a bioluminescence-based cytotoxicity assay was performed (Fig. 3(b) and (c)). PBMCs from healthy donors were incubated with target cells (Ramos, Namalwa) in an E : T ratio of 5 : 1 in presence or absence of the SadP-scFv UCHT1 and luminescence was measured at 7, 24 and 48 hours. Incubation of PBMCs and Ramos cells in presence of increasing concentrations of SadP-scFv UCHT1 led to specific, dose-dependent killing of target cells. Specific killing of Ramos cells was observed 24 hours after incubation, with SadP-scFv UCHT1 triggering between 17% (1 nM) and 42% (10 nM) specific target cell lysis that was increased to 23% (1 nM) and 65% (10 nM) at 48 hours post treatment (Fig. 3(b)). Incubation of Namalwa cells with PBMCs and SadP-scFv UCHT1 did not yield any significant killing of target cells. However, with progressing incubation time and higher lectibody concentrations, around 10% of target cell lysis was observed (10 nM; Fig. 3(c)). As the SadP-scFv UCHT1 lectibody did not affect cell viability in absence of PBMCs (Fig. S8), the observed lysis is thought to be T cell-mediated. Furthermore, similar experiments were performed, in which the SadP-scFv UCHT1 lectibody was compared with the StxB-scFv UCHT1 lectibody in terms of T cell-mediated cytotoxicity (Fig. 3(d) and (e)). Since the StxB-scFv UCHT1 lectibody had previously been used at higher concentrations than the SadP-scFv UCHT1 lectibody, concentrations in the range from 1 to 100 nM were selected in order to test the lectibodies using similar concentrations. The StxB-scFv UCHT1 lectibody was able to mediate cytotoxicity, especially at higher concentrations of ≥25 nM. The SadP-scFv UCHT1 lectibody mediated similar cytotoxicity values at concentrations of ≤10 nM. Both lectibodies were able to mediate a kill rate between 80 and 90% after 48 hours at the highest concentration.

**Fig. 4:**
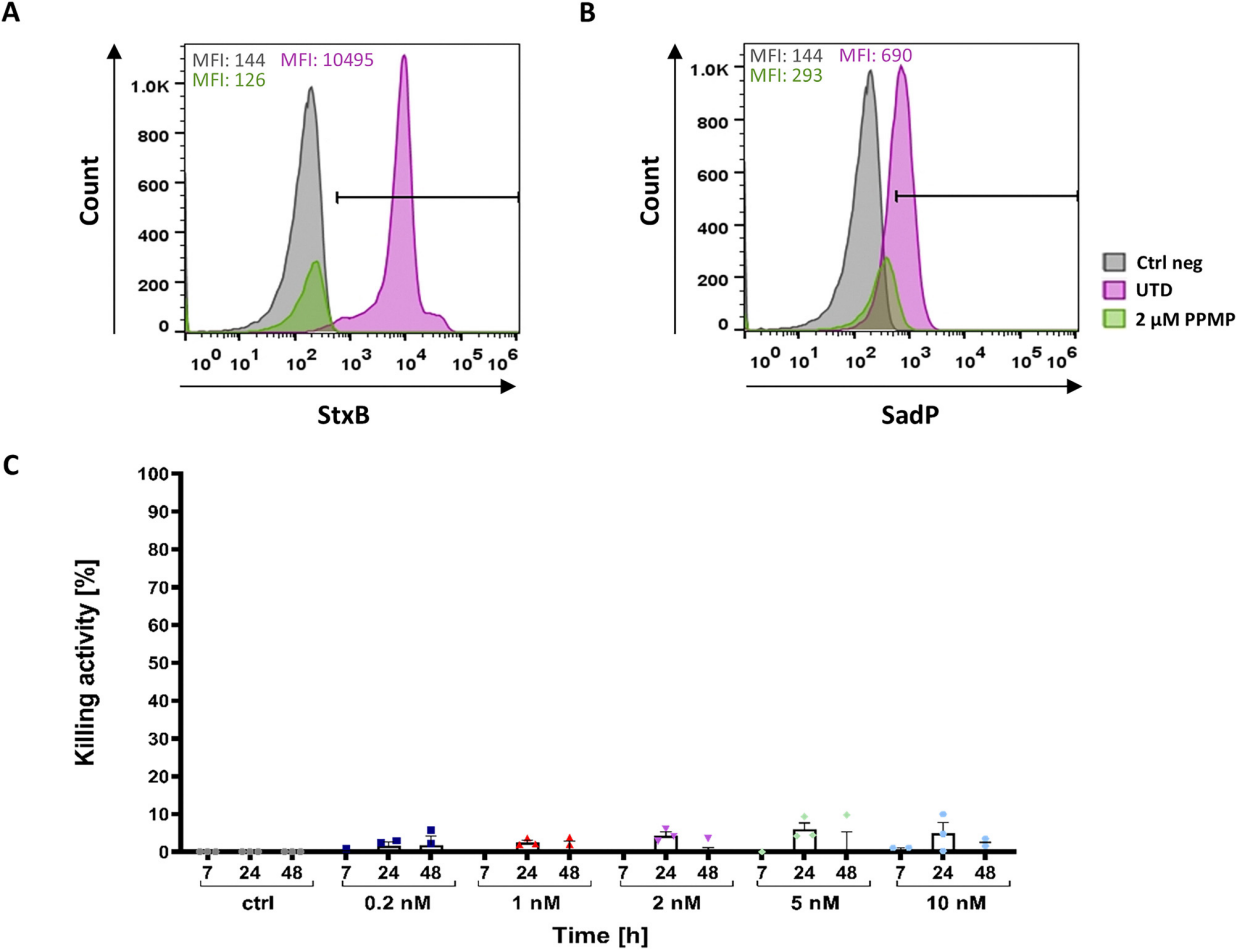
GCS inhibition by PPMP led to abolished target cell lysis. Namalwa cells still exhibit a residual, low Gb3 abundance therefore, Ramos cells were treated with a glucosylceramide synthesis (GCS) inhibitor called PPMP to abolish Gb3 completely. Ramos cells were treated with 2 µM PPMP for 72 hours before starting the incubation with PBMCs and the SadP-scFv UCHT1 treatment. (a), (b) To ensure that the Gb3 was no longer present at the cell surface, the cells were stained with (a) 2.6 nM StxB-AF647 and (b) 20 nM SadP which was detected using an anti-His-Tag-AF647 antibody. Cells receiving no PPMP and remaining unstained were used as a negative control (Ctrl neg). Cells not treated with PPMP (UTD) were stained with either StxB or SadP and used as a positive control. The staining with StxB showed no presence of Gb3 at the cell surface, whereas SadP exhibited a low amount of binding to PPMP-treated Ramos cells. (c) The PPMP treated Ramos cells were incubated as described above with PBMCs and SadP-scFv UCHT1 for 48 hours. No significant killing of target cells could be observed. The data are shown as the mean ± SEM (*N* = 3) of three separate experiments. *n* = 3. Statistical significance was evaluated using a one-way ANOVA. *p*-values < 0.5 were considered significant. *p ≤ 0.05; **p ≤ 0.01; ***p ≤ 0.001; ****p ≤ 0.0001.

As Namalwa cells have a low abundance of Gb3 on the cell surface it is possible that this very low Gb3 content is sufficient to elicit a small amount of lysis of Namalwa cells.^57^ To determine if the low Gb3 amount is leading to the observed target cell lysis, Gb3^+^ Ramos cells were treated with the GCS inhibitor PPMP prior to the bioluminescence-based cytotoxicity assay. The cells were treated with 2 µM PPMP for 72 hours.^59^ Depletion of Gb3 from the cell surface was confirmed by fluorescence staining of treated cells either with an AF647-labelled Shiga toxin B-subunit (StxB-AF647) or with SadP followed by an anti-His-Tag-AF647 antibody. As depicted in Fig. 4(a), binding of StxB-AF647 to Ramos cells was abolished upon treatment with PPMP. However, SadP still exhibited a small amount of residual binding (Fig. 4(b)). It is possible that this binding stems from P1 containing N-glycans being present on the surface of these cells as it is not inhibited by PPMP treatment.^74^ In a bioluminescence-based cytotoxicity assay, the SadP-scFv UCHT1 did not mediate lysis of PPMP-treated Ramos cells (Fig. 4(c)). Moreover, the dose-dependent increase in T cell-mediated target cell lysis observed with Namalwa cells was in this case also abolished (Fig. 4(c)). This suggests that the toxicity towards Namalwa cells that could be seen in Fig. 3(c) was caused by the low amount of Gb3 on the surface of these cells.

### SadP-scFv UCHT1 is able to activate T cells in co-culture with Gb3^+^ target cells

**Fig. 5:**
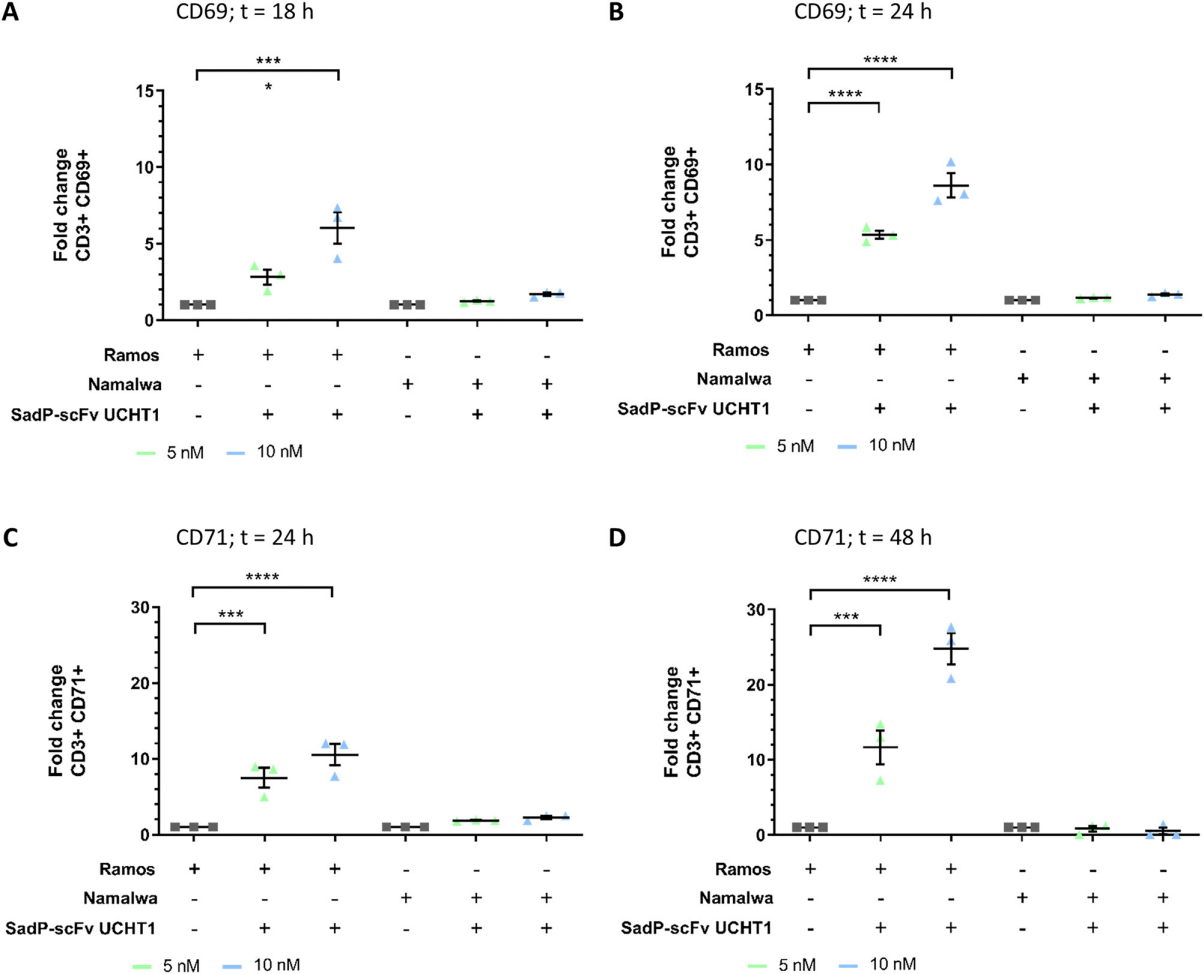
Upregulation of T cell activation markers CD69 (early) and CD71 (middle/late) in presence of the SadP-scFv UCHT1 lectibody. The T cells were incubated with the indicated target cell line and the lectibody. (a) At 18 hours post-incubation, cells were stained with anti-CD3-AF488 (1:200) and anti-CD69-APC (1:200) and analysed using flow cytometry. The MFI of CD3^+^CD69^+^ cells was determined, and the antibody background was subtracted. The Fold increase was calculated from these values and compared to the control (PBMCs together with target cells, without lectibody). Incubation of T cells with Ramos cells and 10 nM lectibody for 18 hours lead to a significant increase in CD69 surface expression of about 6-fold compared to the control. Namalwa cells, on the other hand, did not elicit an increase of CD69 expression on the surface of the T cells in presence of the lectibody. (b) Incubation of PBMCs with SadP-scFV UCHT1 and Ramos cells for 24 hours led to an increase in CD69 fluorescence signal of 5-fold and 8-fold, respectively for both concentrations tested. There was no increase in CD69 signal when incubated with Namalwa cells. (c) After incubating T cells with SadP-scFv UCHT1 and Ramos cells for 24 hours the cells were stained with anti-CD3-AF647 (1:200) and anti-CD71-AF488 (1:200) and analysed by flow cytometry. The CD71 signal was increase significantly ∼7-fold using 5 nM and ∼10-fold using 10 nM SadP-scFv UCHT1. Incubation with Namalwa cells, again did not lead to a significant increase in CD71 signal after 24 hours. (d) Extending the incubation time in presence of Ramos cells to 48 hours resulted in an even greater fold increase (12-fold for 5 nM and 25-fold for 10 nM) while an incubation for 48 hours did not increase the CD71 signal in presence of Namalwa cells. Statistical significance was evaluated using a one-way ANOVA The data are shown as the mean ± SEM (*N* = 3) of three separate experiments. *n* = 3. *p*-Values < 0.5 were considered significant. ***p ≤ 0.001; ****p ≤ 0.0001.

Following an immunological treatment, activation of T cells represents a key event for mediating an immune response. Upon activation, a signalling cascade is set into motion ultimately resulting in the upregulation of activation markers such as CD69 or CD71.^18^ Upregulation or expression of these activation markers follows exact and concise time patterns. Early activation is marked by the rapid expression of CD69 on the T cell surface where it can typically be found between 3 to 12 hours after T cell stimulation. CD69 levels remain elevated for 24 hours and then decrease.^22,23^ CD69 is associated with the CD3 receptor complex and reported to play a role in the proliferation and survival of activated T lymphocytes.^82,83^ The upregulation of CD69 was investigated by co-culturing PBMCs with target cells (Ramos, Namalwa) for the indicated time and then staining the PBMCs with fluorescent anti-CD3-FITC/anti-CD69-AF647 antibodies. PBMCs were gated for CD3 and CD69 double positive cells to gain insight on CD69 upregulations on T cells. PBMCs and target cells (Ramos or Namalwa) in absence of the SadP-scFv UCHT1 were measured as an untreated control and PBMCs incubated without target cells or SadP-scFv but stained with the antibody mixture were added as controls to ensure the fluorescence signal was specifically induced by the presence of SadP-scFv UCHT1 and Gb3^+^ Ramos cells. The fold-increase compared to the untreated control was calculated and the antibody background subtracted. Here, we show a significant 6-fold increase of fluorescence signal of CD69 in T cells upon treatment with 10 nM SadP-scFv UCHT1 after 18 hours of co-cultivation of T cells with Gb3+ Ramos cells (Fig. 5(a)). An incubation with Namalwa cells and the SadP-scFv UCHT1 did not result in an increased CD69 signal on the T cells. This upregulation in presence of Ramos cells and SadP-scFv UCHT1 was further increased at 24 hours of co-cultivation to 5- to 8-fold, for 5 or 10 nM, respectively (Fig. 5(b)).

The transferrin-receptor CD71 is typically found to be upregulated between 24 hours and 72 hours post-activation and therefore marks the mid-to-late stage of T cell activation.^24,83^ CD71 is an iron-transport protein that is associated with the zeta-chain of the TCR. It is an essential factor for proliferating T cells.^84,85^ The assay was performed as described above. PBMCs were gated for CD3 and CD71 double positive cells. When co-incubating PBMCs and target cells in presence of the SadP-scFv UCHT1, CD71 expression on CD3^+^ cells was significantly increased (7-fold for 5 nM, 10-fold for 10 nM) already after 24 hours of co-cultivation with both concentrations tested (Fig. 5(c)). CD71 fluorescence signal was increased after 48 hours of incubation to 12- and 25-fold for 5 and 10 nM, respectively (Fig. 5(d)). These findings underline the efficacy of the SadP-scFv UCHT1 in mediating T cell activation followed by target cell lysis.

## Discussion

The specific targeting of cancer cells is a requirement that any approach to cancer immunotherapy must fulfil. Changes in glycosylation patterns are not only associated with, and sometimes responsible for the onset of tumour progression and proliferation but also with metastasis and invasiveness.^31,33^ Therefore, glycoconjugates offer ideal TAAs to specifically target cancer cells.^87^ In the present study, we aimed to develop a SadP-based lectibody targeting cancer cells characterized by a high abundance of the glycosphingolipid Gb3 on the cell surface. It has been demonstrated that Gb3 is linked to metastasis formation, multi-drug resistance and invasive phenotypes.^33,48,88^ The rational design of the lectibody was inspired by the design of BiTEs. Where in the BiTE format an scFv with affinity for a TAA is genetically linked to an anti-CD3 scFv, the lectibody was designed to exchange the TAA scFv against the monomeric lectin SadP.^77,89^ Like BiTEs, lectibodies cross-link cancer cells with T cells and therefore activates the T cells (Fig. 1(a)). In BiTEs, clustering of the CD3 receptor complexes is responsible for the activation of the T cell without the need of co-stimulatory molecules and the subsequent lysis of the cancer cell.^16^ It can be hypothesised that the activation of T cells through the lectibody happens in the same manner.

As the order of fusion partners is often critical, at least two orientations should be tested.^90^ The peptide signal DsbA (DsbAss) was introduced at the N-terminus for periplasmic export to favour the proper folding of the protein and the formation of disulphide bonds. It is typically cleaved in the export process.^91^ The scFv relies on an oxidizing environment to properly form its disulphide bonds.^60,61^ SadP, which occurs naturally as a membrane-bound protein, is also adapted to cross the periplasm in its native form.^60,90,91^ Therefore, DsbAss-driven periplasmic export is important for the functionality of the protein.^77,89,92^ A small 6× His-Tag was introduced at the C-terminus as it neither interferes with the functionality nor structural property of the protein and facilitates the purification process.^90^ We designed and produced two constructs where the SadP domain was either at the N- or the C-terminus of the scFv part. Only the SadP-scFv UCHT1 was fully characterized as the scFv UCHT1-SadP construct could not be purified in an efficient manner and was only obtained at 50% purity (data not shown).

A key factor in the development of such a fusion protein is the stability and accessibility of the individual protein components in the resulting chimera. In the current study, we used computational modelling to rationally design a fusion protein where SadP and scFv monomer modules were connected by a linker of appropriate length and flexibility. The desired monomeric SadP-scFv UCHT1 fusion protein was constructed *in silico*, and both the folding and the linker length were suitable to retain the binding sites assessable for their receptors. The lectibody was proven to be stable in the course of MD trajectory. In the StxB-based lectibody version, developed by Tomisch *et al.*,^58^ a stable interaction between the scFv UCHT1 and the StxB due to hydrophobic patches on the surface of the StxB dimer was observed. These interactions most likely resulted in only one operational scFv UCHT1 as the other scFv present in this construct was permanently needed, to stabilize the fusion protein.^58^ In the SadP-scFv UCHT1 this phenomenon cannot be observed due to a shallow energy surface which is driving the protein to keep a compact shape while at the same time remaining flexible.

Heterologous expression in *E. coli* requires to determine the best expression conditions by varying inductor concentration, induction temperature and length. The best yield of the lectibody was obtained for an expression of 18 hours at 20 °C and an induction with 1 mM IPTG (Fig. S5). The expression of recombinant proteins in *E. coli* is recommended to be carried out between 15 and 25 °C since lowering expression temperature is advantageous to proper protein folding.^92^ At lower temperatures, protein expression is indeed slowed down allowing a better support from the chaperones leading to less unfolded/misfolded proteins or aggregates.^92–94^ The previously developed StxB-based lectibody^58^ exhibited some contaminants after the IMAC affinity purification resulting in some unspecific binding. The StxB-based lectibody also had a very low yield (0.2 mg L^−1^). When developing the SadP-scFv UCHT1 lectibody, the periplasmic export was therefore guided by the DsbAss instead of pelB. With DsbAss the periplasmic export happens co-translationally^91^ whereas with pelB it happens post-translationally.^95,96^ This could potentially provide an explanation for the near absence of contaminants in SadP-scFv UCHT1 compared to the StxB-scFv UCHT1 version. The SadP-based lectibody also exhibited an improved yield (3 mg L^−1^).

Gb3 is overly abundant in many different types of cancer like breast-, ovarian-, colorectal cancer and Burkitt's lymphoma. It is linked to several factors worsening prognosis of cancer patients.^47,97–99^ Burkitt's lymphoma was chosen as a model in the present study.

The SadP-scFv UCHT1 lectibody did not affect viability of target cells without the presence of PBMCs (Fig. S8). It demonstrated specific, dose-dependent binding towards Gb3 on cancer cells and to the CD3 receptor on T cells. At the same time, off-target binding to Namalwa cells was abolished (Fig. 3(a)). The SadP-scFv UCHT1 lectibody was able to redirect T cells to lyse Gb3^+^ Ramos cells (65% target cell lysis for 10 nM lectibody; Fig. 3(b)). Lectibody-induced cell lysis was abolished when SadP-scFv UCHT1 and PBMCs were incubated together with Gb3^−^ Namalwa cells, or Gb3-depleted Ramos cells (∼10% residual killing with Namalwa cells; Fig. 3(c) and 4(c)). One possible explanation for this unspecific killing could be traces of endotoxins remaining in the protein sample after purification, which could activate T cells independently of lectibody binding. Therefore, the endotoxin concentration was determined in both the SadP-scFv UCHT1 and StxB-scFv UCHT1 lectibody solutions. For the SadP-scFv UCHT1 lectibody, an endotoxin concentration of 0.5 endotoxin units per ml was determined at a lectibody concentration of 1 nM. For the StxB-scFv UCHT1 lectibody, a 10-fold higher endotoxin concentration was measured. These endotoxin levels would explain the low but not negligible cytotoxicity towards Gb3^−^ Namalwa cells. An additional purification step to remove endotoxins could potentially reduce this unspecific killing. For future studies and *in vivo* applications, the production of lectibodies in mammalian cell lines is an important next step. Compared to *in vitro* studies, *in vivo* studies will provide an even better understanding of how these bispecific protein constructs influence more complex environments.

When comparing the SadP-scFv UCHT1 with the StxB-lectibody formats published by Rosato *et al.* (clicked construct), and Tomisch *et al.* (fusion construct), it becomes evident that the SadP-scFv UCHT1 was able to induce similar killing activity as the StxB-scFv UCHT1 at the highest concentration used of 100 nM. SadP-scFv UCHT1 also induced significant killing activity at concentrations as low as 1 nM, while StxB-scFv UCHT1 induced significant killing after 48 hours at concentrations of 10 nM and higher. A comparison of the binding of SadP-scFv UCHT1 and previously published lectibody constructs further supports this observation. At 10 nM, SadP-scFv UCHT1 showed 35% higher binding to Gb3^+^ cells and only about 5% less binding than the StxB-scFv OKT3.^58,59^ SadP has an affinity towards Gb3 of ∼13 µM,^60^ while the one of monomeric StxB is thought to be ∼0.5 mM.^58,100^ StxB in its native pentameric form however, has an avidity for Gb3 of 0.22 µM.^101^ In the StxB-scFv UCHT1 the StxB though was dimeric and hence had a reduced avidity of ∼0.5 mM.^58,100^ Due to its simpler structure and the fact that no oligomerisation is required, SadP in principle is a more suitable candidate for the production of genetically linked lectibodies. This increased affinity of SadP-scFv UCHT1 relative to StxB-scFv UCHT1 led to lower concentrations needed for sufficient target cell lysis. Transferring this to possible *in vivo* studies a lower dosage would potentially mean fewer off-target effects.^102,103^ A reduced T cell-mediated target cell lysis compared to the StxB-scFv OKT3 (∼5%) could also be attributed the higher number of scFv in that construct. Here, one scFv is linked to a SadP monomer, whereas the clicked StxB-scFv OKT3 lectibody exhibited three scFvs per pentamer of StxB.^59^

The SadP-scFv UCHT1 lectibody has one drawback in comparison with the StxB-based lectibodies, which is its recognition of Gb3 *via* the terminal Galα1–4Gal moiety without needing the glucose moiety.^53^ This leads to off target recognition of Gb4, but possibly also the P1 blood-group antigen, which also exhibits a terminal Galα1–4Gal and can be found on *N*-glycans.^60,104,105^ The P1 blood-group antigen and Gb3 are synthesized by the α1,4-galactosyltransferase (pA4GalT). Unlike Gb3, however P1 synthesis is not inhibited by treating cells with PPMP.^74^ However, the *in vitro* assay performed with PPMP-treated Ramos cells shows, that a depletion of Gb3 was sufficient to abolish target cell lysis (Fig. 4(c)). When considering moving *in vivo*, it is important, though to have a strict affinity for the TAA. Therefore, it would be necessary to mutate the binding site of the SadP to abolish Gb4 and P1 binding.^53,60,61^ An additional approach, promising high avidity is the combination of multiple low affinity binders to achieve high avidity and selective targeting of cells overexpressing the TAA. This approach has been shown to work previously in a HER2/CD3 antibody and a peptibody format.^106,107^ It could therefore be beneficial to make a trivalent, genetically linked lectibody, combining two SadP with one anti-CD3 scFv and therefore gaining a low affinity but high avidity molecule potentially showing improved cytotoxicity at lower picomolar dosage.

The cross-linking of T cells to cancer cells mediated by the SadP-scFv UCHT1 lectibody resulted in T cell activation, both CD4^+^ and CD8^+^. T cell activation is a tightly regulated cascade and the activation status of the T cell can be pinpointed by measuring certain markers on the cell surface by flow cytometry.^108^ In this study, we used a physiological mixture of CD4^+^ and CD8^+^ T cells derived from peripheral human blood donations as the lectibody targets the CD3 receptor and it can employ its receptor function on both CD4^+^ and CD8^+^ T cells.^89^ An early activation of T cells was confirmed by CD69 expression on the surface of T cells. According to literature, CD69 expression is found to be upregulated between 3–12 hours following T cell stimulation and remains high up to 24 hours.^22,23^ CD71 is expressed in resting T cells and is found to be upregulated 24–72 hours post-stimulation, making it a mid-to-late stage activation marker.^84,86^ BiTE-induced CD3 clustering was found to prompt the upregulation of CD69 on T cells.^10,78^ Surface expression of CD69 and CD71 was measured 18 hours to 48 hours after addition of the SadP-scFv UCHT1. The lectibody was able to induce an increase of CD69 on T cells within 18 hours. Previous studies using different BiTEs showed an increase of CD69 after 24 to 48 hours.^78,109–112^ The EpCAM/CD3 BiTE MT110 revealed an elevation of CD69 signal after 24 hours at doses ≤14.5 pM and found no significant difference in upregulation between CD4^+^ and CD8^+^ T cells.^78,109^ A similar trend was observed by Brandl *et al.* upon treatment with ∼45 pM of the CD19/CD3 BiTE MT103, where an increase of CD69 signal was recorded after 24 hours and decreased afterwards. Both CD4^+^ and CD8^+^ T cells demonstrated an increased CD69 signal.^110^ The lectibody StxB-scFv OKT3 induced an increase of CD69 on the surface of T cells after 24 hours.^59^ The activation marker CD71, pinpointing mid-to-late stage activation^24^ was also found to be increased after incubation of PBMCs with Gb3^+^ Ramos cells and the SadP-scFv UCHT1 after about 24 hours to 48 hours. The expression of CD71 in CD8^+^ T cells treated with 18 pM of the CD33/CD3 BiTE AMG330 was found to be increased after 24 hours reaching its peak values after 72 hours.^111,112^ For DLL3/CD3 BiTE AMG757, the CD71 signal was increased at 48 hours post-treatment, with no significant difference between CD4^+^ and CD8^+^ T cells.^112^ When investigating T cell activation mediated by the StxB-scFv OKT3 Rosato *et al.* found an increase of CD71 between 24 and 48 hours.^59^ The observations made in the present study are in accordance with these reported time points. The observed cell death and the activation by the lectibody is a result of a variable sub-population of T cells that are naturally present and vary between donors.^113^

## Conclusion

To summarize, the SadP-scFv UCHT1 lectibody demonstrates improved efficacy towards the elimination of Gb3^+^ cancer cells compared to previous lectibody formats targeting Gb3^+^ cancer cells. The SadP-based lectibody was able to induce specific target cell lysis in Gb3^+^ Burkitt's lymphoma cells. Activation of the T cells after incubation with target cells and low nanomolar concentrations of SadP-scFv UCHT1 could be shown. The presence of the monomeric SadP as opposed to oligomeric lectins, like StxB, greatly improves the yield as it forgoes multimerization and makes it easily up scalable. The efficacy of this lectibody treatment remains to be proven in *in vivo* studies. Yet, this study highlights again the great potential that lectins provide to specifically target cancer cells *via* their aberrant glycosylation patterns.

## Ethics approval and consent to participate

The LRS chambers used as PBMC sources were purchased from the blood bank of the University Medical Centre Freiburg (approval of the University Freiburg Ethics Committee: 147/15).

## Author contributions

Conceptualization, J. T., F.R. and W. R.; methodology, J. T., J. G., O. N. M., S. F. M, A. V., and W. R.; formal analysis, J. T.; investigation, J. T., J. G., O. N. M., P. S., data curation, J. T.; writing—original draft preparation, J. T.; writing—review and editing, J. T., O. N. M., F. R., J. G., A. V., A. I., P. S., S. F. M. and W. R.; visualization, J. T., O. N. M.; supervision, W. R.; project administration, J. T. and W. R.; funding acquisition, W. R. All authors have read and agreed to the submitted version of the manuscript.

## Conflicts of interest

The authors declare no conflict of interest.

## Abbreviations

ADC: Antibody–drug conjugate
AF647: Alexa Fluor 647
ALL: Acute lymphoblastic leukaemia
AML: Acute-myeloid leukaemia
BiTE: Bispecific T cell engager
FDA: Food and drug administration
Fhb: Factor H binding protein
Gb3: Globotriaosylceramide
GC: Germinal centre
GCS: Glucosylceramide synthase
GSL: Glycosphingolipid
IMAC: Immobilized metal affinity chromatography
MD: Molecular dynamics
MHCI: Major histocompatibility complex I
MM–PBSA: Molecular mechanics Poisson–Boltzmann surface area
OD: Optical density
OS: Overall survival
pA4GalT: α1,4-Galactosyltransferase
PBMCs: Peripheral blood mononuclear cells
PME: Particle Mesh Ewald
PPMP: d-l-*Threo*-1-phenyl-2-palmitoylamino-3-morpholino-1-propanol
R/R: Relapsed and/or refractory
SadP: Streptococcal adhesin P
scFv: Single chain variable fragment
StxB: Shiga toxin B-subunit
TAA: Tumour-associated antigen
TACAs: Tumour-associated carbohydrate antigens
TCR: T cell receptor
VH: Heavy chain
VL: Light chain
WHO: World Health Organisation

## Supplementary Material

CB-OLF-D5CB00027K-s001

## Data Availability

Raw data available upon reasonable request from the corresponding author. The data used in the manuscript is either included in the main file or provided in the SI. Supplementary information is available. See DOI: https://doi.org/10.1039/d5cb00027k.
