# Supplementary material for: A novel SadP-scFv UCHT1 lectibody activates T cells and mediates lysis of Burkitt's lymphoma cells
Source: RSC Chem Biol. 2025 Nov 20;7(1):81–97. doi: 10.1039/d5cb00027k (PMC12666658; doi:10.1039/d5cb00027k)
Supplement: CB-007-D5CB00027K-s001 [file CB-007-D5CB00027K-s001.pdf]

Supplementary Information (SI) for RSC Chemical Biology.
This journal is © The Royal Society of Chemistry 2025

A novel SadP-scFv UCHT1 lectibody activates T cells and mediates lysis of Burkitt's lymphoma cells

Jana Tomisch [1,2], Jonas Gräber [1,2], Olga N. Makshakova [1,2], Pavel Salavei [2,3], Francesca Rosato [1,2], Sarah Frisancho Mariscal [1,2], Annabelle Varrot [4], Anne Imberty [4], Winfried Römer [1,2*]

[1] Faculty of Biology, University of Freiburg, 79104 Freiburg, Germany

[2] Signalling Research Centres BIOSS and CIBSS, University of Freiburg, 79104 Freiburg, Germany

[3] Core Facility Signalling Factory & Robotics, University of Freiburg, 79104 Freiburg, Germany

[4] Université Grenoble Alpes, CNRS, CERMAV, 38000 Grenoble, France

*** Correspondence:** winfried.roemer@bioss.uni-freiburg.de

Jana Tomisch:	jana.tomisch@bioss.uni-freiburg.de; ORCID: 0000-0002-4656-9345
Jonas Gräber:	jonas.graeber@bioss.uni-freiburg.de
Olga N. Makshakova:	olga.makshakova@biologie.uni-freiburg.de; ORCID: 0000-0002-0615-3513
Pavel Salavei:	pavel.salavei@bioss.uni-freiburg.de
Francesca Rosato:	francesca.rosato.bis@gmail.com; ORCID: 0000-0002-9255-9391
Sarah Frisancho Mariscal:	sarah.frisancho@med.uni-muenchen.de
Annabelle Varrot:	annabelle.varrot@cermav.cnrs.fr; ORCID: 0000-0001-6667-8162
Anne Imberty:	anne.imberty@cermav.cnrs.fr; ORCID: 0000-0001-6825-9527
Winfried Römer:	winfried.roemer@bioss.uni-freiburg.de; ORCID: 0000-0002-2847-246X

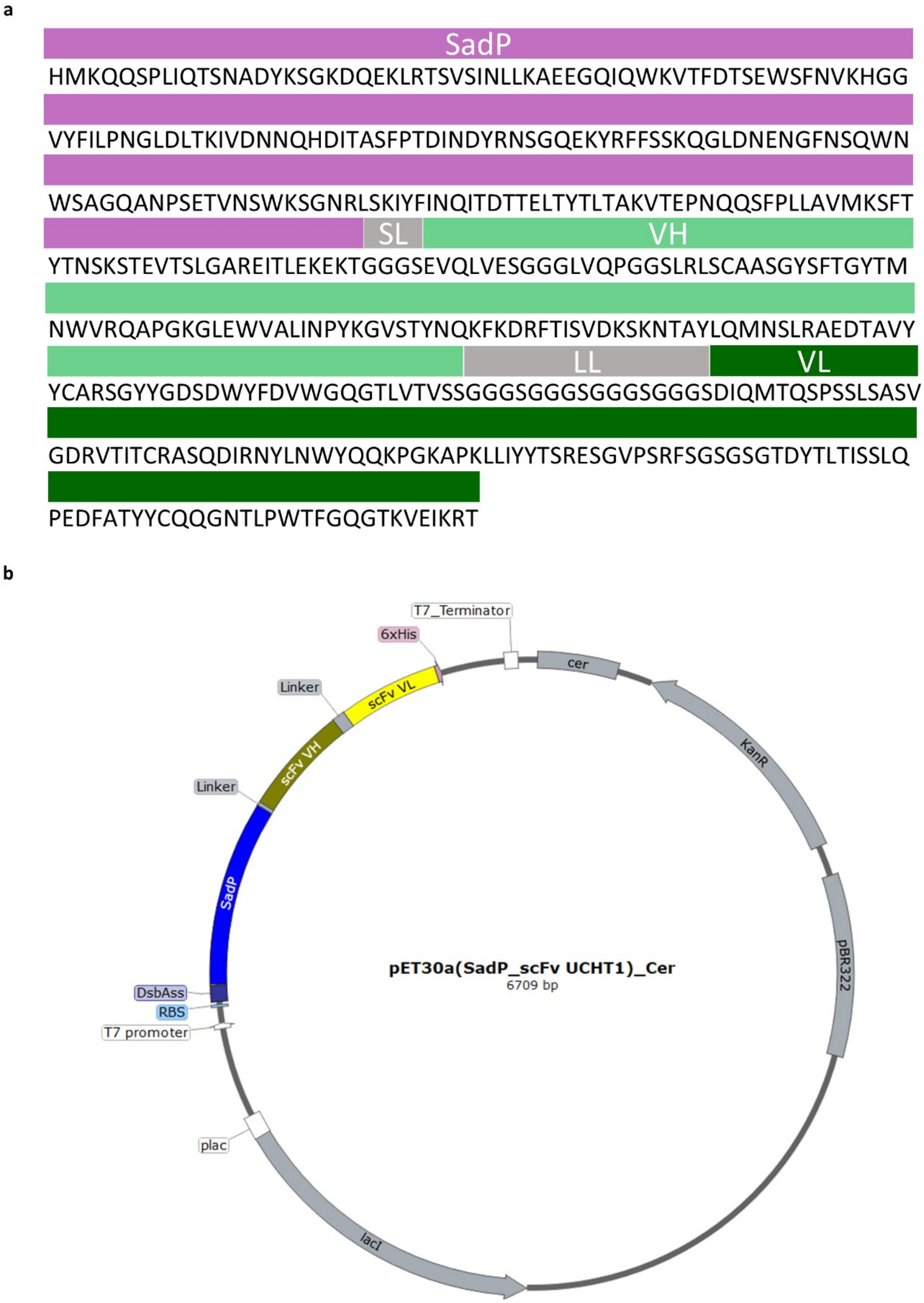

Supplementary Figure S1 Sequence and plasmid map of the SadP-scFv UCHT1 lectibody. (a) Amino acid sequence of the SadP-scFv UCHT1 lectibody. The Sequence of SadP (purple) is linked to the heavy chain (VH, light green) of the scFv UCHT1via a short 1x GGGS linker (grey). The VH is followed by the light chain of the scFv UCHT1 (VL, dark green). The chains are connected via a 4x GGGS linker. (b) The plasmid contains the sequence of SadP linked to the αCD3 VH via a 1x GGGS linker and the αCD3 VL via a 4x GGGS linker followed by a 6x HisTag used for purification. The DsBA signalling sequence (DsbAss) included in the plasmid ensures the translocation of the fusion protein into the periplasm. The expression of the recombinant fusion protein is under the control of the lac operator. The lacI gene encodes the lac repressor, it represses lac operator and therefore hindering gene expression. This repression can be relieved by addition of IPTG. The plasmid also includes a kanamycin resistance (KanR) for antibiotic selection. A cer site is included to increase plasmid stability.

a b

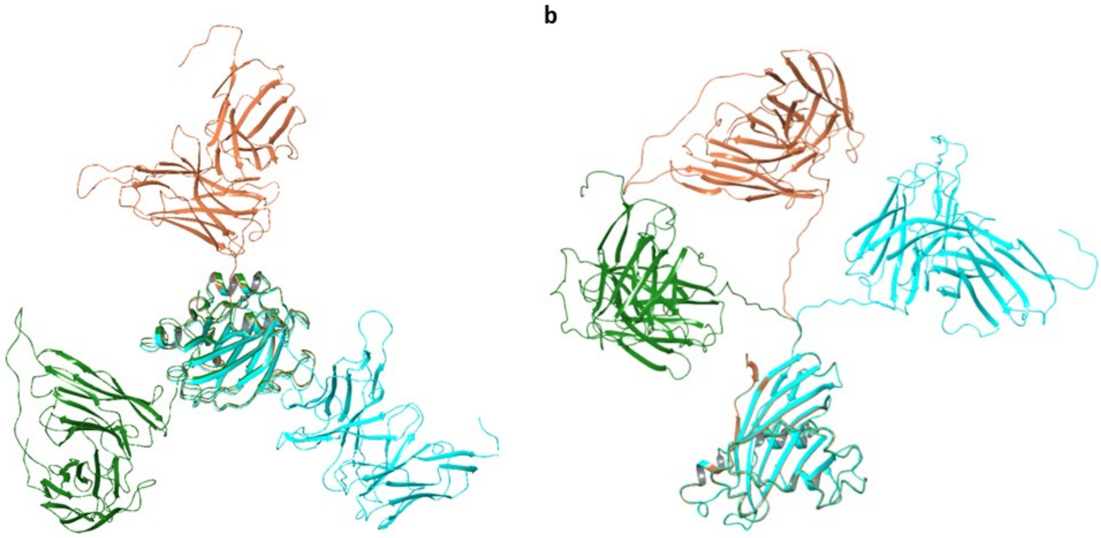

Supplementary Figure S2 Enhanced conformational sampling of three additional structures. The scFv UCHT1 was systematically positioned in respect to SadP. (**a**) Side view and (**b**) top view of three initial conformations of the SadP-scFv UCHT1 lectibody (depicted in brown, cyan and green) that were used for the MD equilibration. The scFv UCHT1 structure of the SadP-scFv UCHT1 fusion protein was built up using Modeller9.15. The scFv UCHT1 structure was built up by homology modelling based on the Diabody 31 (PDB code: 6KR0) structure. Moreover, the x-ray structure of SadP (PDB code: 5BQA)

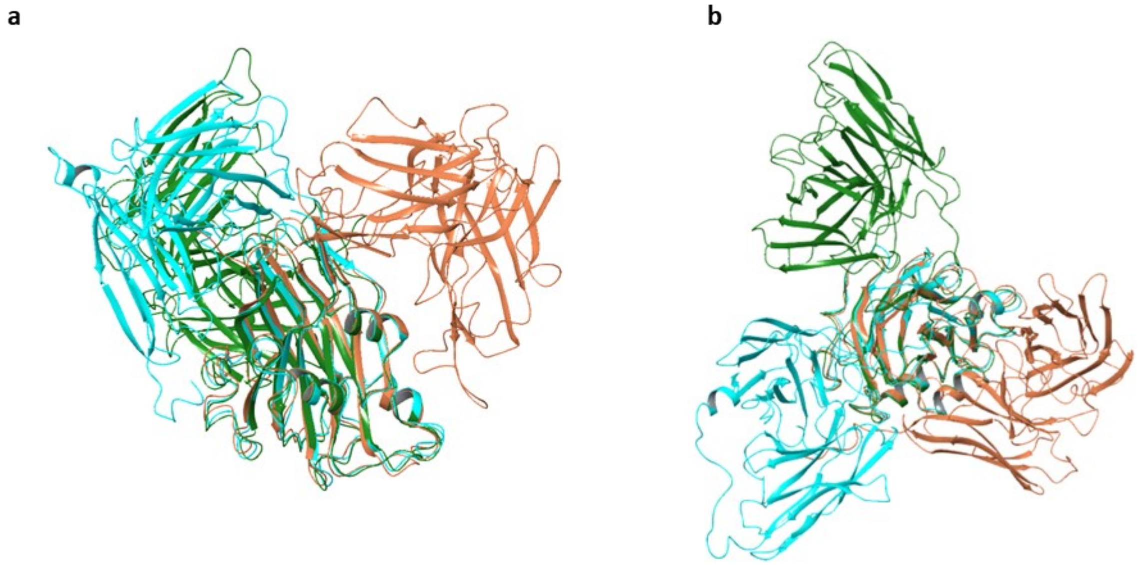

Supplementary Figure S3 Determination of the dynamic character of SadP-scFv UCHT1. To determine the dynamic character of the lectibody, the three initial structures (those shown in Figure S1) were equilibrated during the course of MD trajectories. (**a**) Side view and (**b**) top view of the three structures of SadP-scFv UCHT1 lectibody, equilibrated in the course of 1 μs MD trajectories. The color-coding (brown, cyan, green) relates to a single chain and correlates to the colours used in Figure S1. Three structures were superimposed on SadP. These structures revealed a tendency of the lectibody to compact, and to present a large surface area of SadP for interactions with the scFv UCHT1.

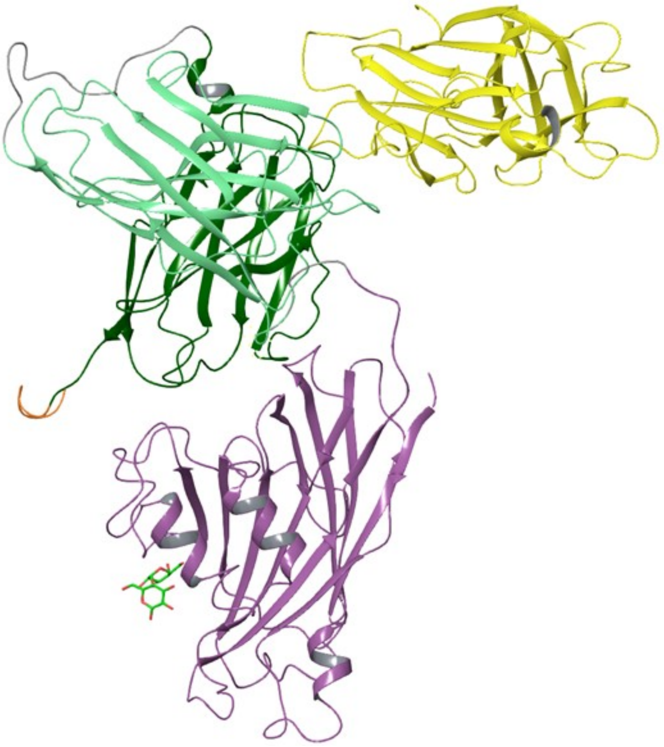

Supplementary Figure S4 Equilibration of the SadP-scFv UCHT1 lectibody in the course of trajectory no. 1. A model of the SadP-scFv UCHT1 lectibody was equilibrated in the course of trajectory no. 1 showing potential interactions with D-galactose (copied from pdb: 5BOA) and CD3 (copied from pdb: 1SY6). In the lectibody, the Gb3 and CD3 binding sites were available for interactions with their respective receptors. Colour code: SadP - violet; scFv VH - light green; scFv VL - dark green; CD3 -

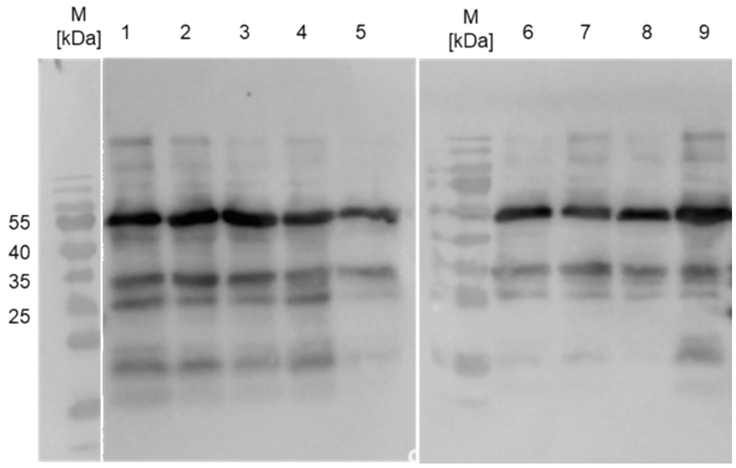

Supplementary Figure S5 Determination of the optimal expression conditions for the SadP-scFv UCHT1 lectibody. To determine the combination of expression temperature and amount of IPTG, the crude lysate of the cytoplasmic fraction was loaded onto a 12% gel, the proteins were separated according to size, transferred onto a WB membrane and stained with an anti-His-Tag-HRP antibody. The expression temperature was varied between 20°C (lane 1-3), 25°C (lane 4-6) and 30°C (lane 7-9) and combined with either 0.1 mM (lane 1, 4 and 7), 0.5 mM (lane 2, 5 and 8) or 1 mM (lane 3, 6 and 9) of IPTG. It was determined that the combination of an overnight (18 hours) expression at 20°C with an expression induction of 1 mM IPTG achieved the highest yield.

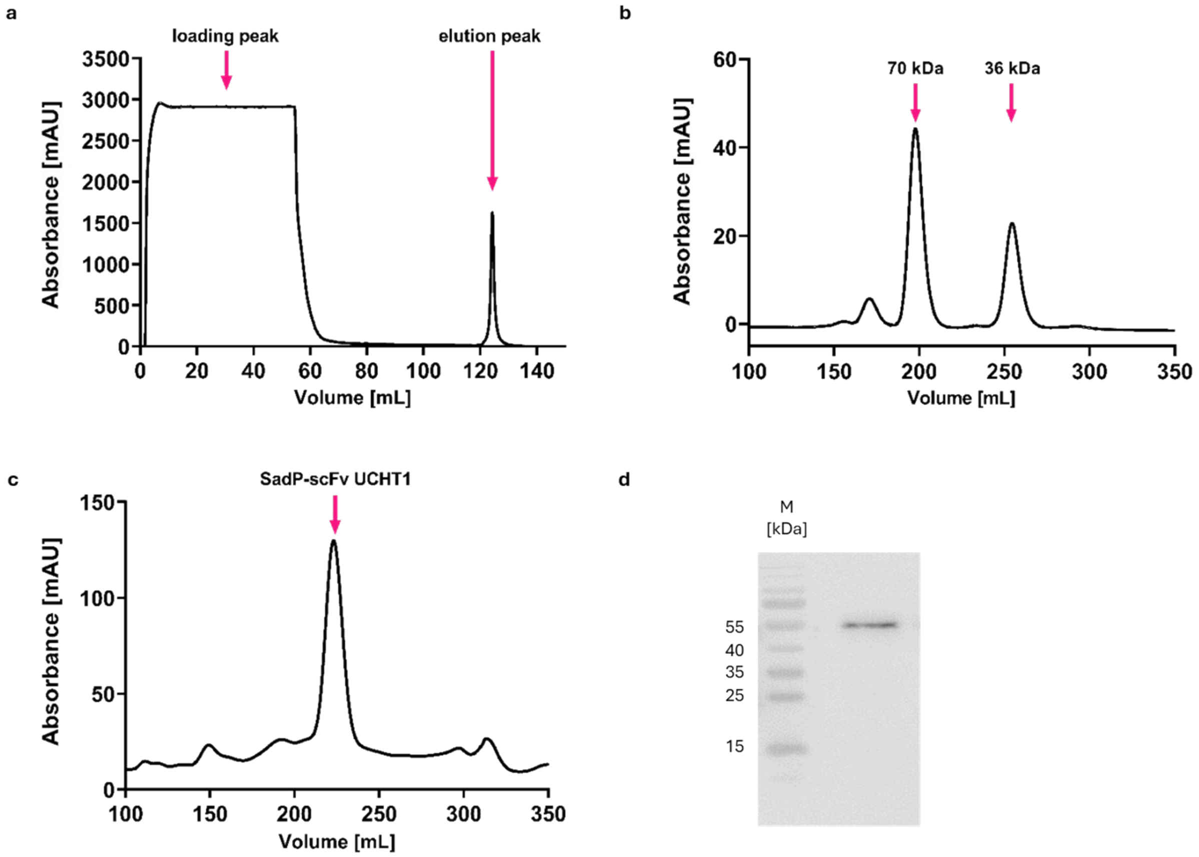

Supplementary Figure S6 Chromatograms for the IMAC affinity purification and size exclusion chromatography of SadP-scFv UCHT1 in comparison to known proteins to determine protein size. (**a**) The lysate was loaded onto a HisTrap 5 mL column for IMAC affinity purification (loading peak). Afterwards the column was washed, and the protein was eluted (elution peak) using an elution buffer containing 500 mM imidazole. (**b**) To determine the size of a protein, the elution volume was compared to that of proteins with a known size. Here we used the previously published StxB-scFv UCHT1 peaks 2 and 3. Peak 2 has a size of ~70 kDa and peak 3 has a size of ~36 kDa. (**c**) As the SDS-PAGE with subsequent immunoblot showed the presence of contaminants a size exclusion chromatography was performed to obtain a pure form of the SadP-based lectibody. The SadP-scFv UCHT1 has a theoretical size of 52 kDa, the elution volume should be approximately 220 mL. The lectibody peak could be found at ~220 mL therefore confirming the theoretical protein size. (**d**) SDS-PAGE/WB showing the lectibody after the size exclusion. The SadP-based lectibody was pure after SEC.

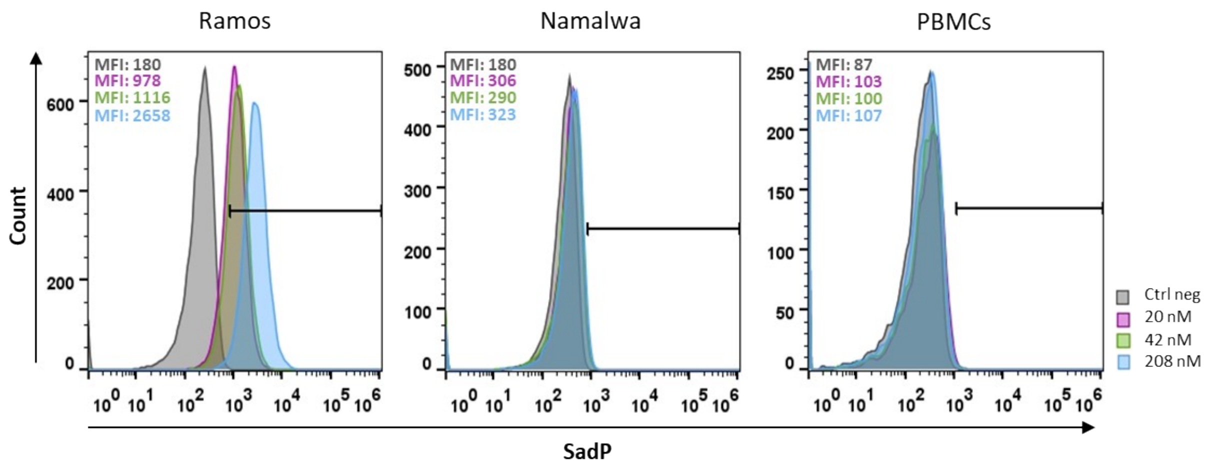

Supplementary Figure S7 Binding of SadP to target and effector cells. SadP was incubated with the cells for 30 minutes, on ice and then stained with an anti-HisTag-AF647 antibody. Ramos cells, with a high Gb3 content, showed a strong SadP binding, while Namalwa cells, with a very low Gb3 content, showed only minimal binding of SadP. No binding of SadP to PBMCs could be observed.

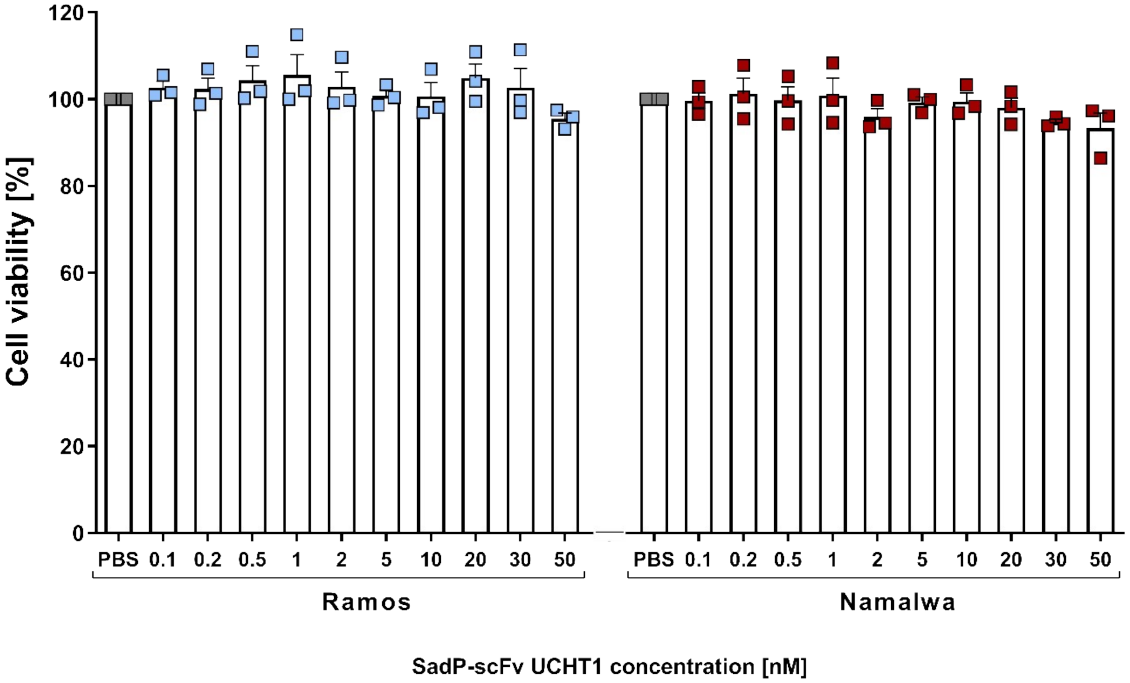

Supplementary Figure S8 Cell viability assay (MTT) to determine possible T cell independent toxicity of the SadP-scFv UCHT1 lectibody. Ramos and Namalwa cells were seeded and incubated with various amounts of SadP-scFv UCHT1 (0.1, 0.2, 0.5, 1, 2, 5, 10, 20, 30, 50 nM) for 24 hours. There were no significant cytotoxic effects detectable caused solely by the presence of the SadP-scFv UCHT1 lectibody. Cell viability was found to be above 85 %. The data are shown as the mean ± SEM (N = 3) of three separate experiments. n = 3.

Supplementary Table S1 Binding energies between SadP and scFv UCHT1 as parts of the lectibody estimated in the course of four MD trajectories. Trajectory no. 1 was initiated from the structure derived from docking (Figure 2 b), trajectories no. 2, 3 and 4 were initiated from the structures shown in Supplementary Figure S1.

Number of trajectory	MMGBSA binding energy, kcal/mol
1	-18 ± 33.5
2	-19 ± 46.9
3	-13 ± 36.2
4	-21 ± 42.6
